# Gut Microbiota Patterns Predicting Long-Term Weight Loss Success in Individuals with Obesity Undergoing Nonsurgical Therapy

**DOI:** 10.3390/nu14153182

**Published:** 2022-08-03

**Authors:** Stephan C. Bischoff, Nguyen K. Nguyen, Benjamin Seethaler, Julia Beisner, Philipp Kügler, Thorsten Stefan

**Affiliations:** 1 Institute of Nutritional Medicine, University of Hohenheim, 70593 Stuttgart, Germany; b.seethaler@uni-hohenheim.de (B.S.); julia.beisner@uni-hohenheim.de (J.B.); 2 Microbiome Insights Inc., Vancouver, BC V6R 4K6, Canada; nguyenkhoibiotech@gmail.com; 3 Institute of Applied Mathematics and Statistics & Computational Science Lab, University of Hohenheim, 70593 Stuttgart, Germany; philipp.kuegler@uni-hohenheim.de (P.K.); thorsten.stefan@uni-hohenheim.de (T.S.)

**Keywords:** microbiota, microbiome, weight loss, prediction, machine learning, obesity

## Abstract

*Background*: The long-term success of nonsurgical weight reduction programs is variable; thus, predictors of outcome are of major interest. We hypothesized that the intestinal microbiota known to be linked with diet and obesity contain such predictive elements. *Methods*: Metagenome analysis by shotgun sequencing of stool DNA was performed in a cohort of 15 adults with obesity (mean body mass index 43.1 kg/m^2^) who underwent a one-year multidisciplinary weight loss program and another year of follow-up. Eight individuals were persistently successful (mean relative weight loss 18.2%), and seven individuals were not successful (0.2%). The relationship between relative abundancies of bacterial genera/species and changes in relative weight loss or body mass index was studied using three different statistical modeling methods. *Results*: When combining the predictor variables selected by the applied statistical modeling, we identified seven bacterial genera and eight bacterial species as candidates for predicting success of weight loss. By classification of relative weight-loss predictions for each patient using 2–5 term models, 13 or 14 out of 15 individuals were predicted correctly. *Conclusions*: Our data strongly suggest that gut microbiota patterns allow individual prediction of long-term weight loss success. Prediction accuracy seems to be high but needs confirmation by larger prospective trials.

## 1. Introduction

Obesity has become a worldwide problem that requires substantial improvement of both prevention and therapy in children and adults [1]. While bariatric surgery offers a sustained therapy of obesity—albeit not without risks and side effects—nonsurgical therapy is often not lasting [2,3]. The latter can be also effective, especially if initiated using a formula-based, low-calorie diet (LCD), but midterm weight gain occurs very often if no weight maintenance activities happen [4]. In a database containing data from 8296 patients with obesity from a multicenter clinical trial, LCD-based intervention performed across Germany, we found that a mean relative weight loss (RWL) of 20.4% and a mean excess weight loss of 52.5% can be achieved after 6 months [5]. However, approximately 18% of the participants did not achieve a relative weight loss of >10% after one year (per protocol analysis) and about 71% experienced significant weight gain after three years without further intervention [5]. Thus, initial success is great, but long-term success is moderate in most cases, indicating the need for long-term maintenance strategies and reliable predictor variables of success. The analysis also shows that the outcome of such weight reduction programs is variable, and determinants of the outcome are of major interest, among which the intestinal microbiota might play a role [3,6].

In a previous trial [7], we studied intestinal microbiota changes in fecal samples from individuals with obesity undergoing a nonsurgical weight loss program by using whole metagenome shotgun sequencing. Microbiota data were analyzed in relation to anthropometric and metabolic data over the course of two years. We found that the microbiota pattern changed in response to the dietetic and lifestyle intervention but tended to return to the initial situation, both at the taxonomical and functional level, at the end of the one-year-long intervention, except for an increase in *Akkermansia* abundance, which remained stable for two years. The *Firmicutes/Bacteroidetes* ratio was higher in subjects with obesity with metabolic syndrome than in the so-called “healthy obese”. Most interestingly, participants who succeeded in losing their weight consistently over the two years had a different microbiota pattern at baseline compared to patients who were less successful in weight reduction [7]. Therefore, we hypothesized that specific microbiota patterns could predict weight loss success. Similar approaches have been made in the past; however, the observation methods were quite short in most cases (between 1–6 weeks) and the data were heterogeneous [8]. Here, we intensified our analyses by applying different predictive statistical techniques. This methodology may allow weight-loss prediction according to the abundance of only a few selected bacterial species or genera, which could be quantified by straightforward qPCR technique in routine settings. Our principal aim was to identify a set of most important bacterial predictor variables allowing for weight-loss prediction on an individual basis.

## 2. Materials and Methods

### 2.1. Weight-Loss Intervention Trial

For the present study, we selected 15 subjects according to defined criteria (see below) out of a larger cohort of adults with obesity with a mean body mass index (BMI) of 42.4 ± 6 kg/m^2^ and age of 40 ± 8 years (Table 1) from a multicenter clinical trial (ClinicalTrials.gov identifier: NCT01344525), approved by the ethics committee of the University Hospital of Tübingen, Germany. The study was conducted at the Optifast^®^52 center at the Metabolic Unit of the University of Hohenheim (Stuttgart, Germany), including adults who lived in the Stuttgart area. Advertisement for the Optifast^®^52 program in Stuttgart was displayed in local newspapers, on billboards, and on social media. All individuals included volunteered to participate in the present study after they received detailed written information about the study and its purpose, as well as personal informative meetings with the Optifast^®^52 team if desired. The primary endpoint of the study was weight-loss maintenance. The secondary endpoints comprised quality of life, as well as anthropometric and biological assessments, as described in detail in the study registration on ClinicalTrials.gov. The study was conducted according to the declaration of Helsinki. Written informed consent was obtained from every subject. Study details and methodology have been described elsewhere [7]. Briefly, exclusion criteria were gastrointestinal disease, severe eating disorders, and treatment with anti-, pre-, or probiotics within three months before sample collection. Selection criteria included a similar BMI (around 40 kg/m^2^) and mid-age (30–50 years) at baseline, and a subject’s affiliation to the same enterotype (Bacteroides-enterotype, determined through sequencing of the baseline sample) to minimize interindividual variability. Among those who fulfilled these criteria, we selected 8 individuals who were highly successful regarding weight-loss maintenance after two years (RWL at ≥10%, persistent success = PS group), and 8 matched pairs who were not successful (RWL < 10%, nonpersistent success = NS group). One had to be excluded from analysis because microbiota analysis failed. A threshold of 10% weight loss and maintenance of it for at least one year has been proposed as the definition for successful weight-loss maintenance [9]. In fact, the RWL was on average 18.2% in the PS group and 0.2% in the NS group [7].

After inclusion into the study, all participants underwent a defined and highly effective multidisciplinary weight loss program (Optifast^®^52, Nestlé Health Science Germany GmbH, Frankfurt, Germany) described before [5]. Briefly, it consisted of a multidisciplinary lifestyle modification over 52 weeks based on four modules (medicine, psychology, exercise, and dietetics). Medical examinations comprised extensive individual medical monitoring by physicians throughout the program. Psychological counseling comprised individual and group sessions with a psychologist focusing on behavioral training and expectation management. Professional sport coaches led group sessions tailored to overweight and obese individuals. Furthermore, individualized advice was given about how to improve physical activity in daily routine. Dietary intervention included the use of an LCD (800 kcal/day) offered as a formula diet for 12 weeks, a switch phase from formula to normal diet for another 12 weeks, and a consolidation phase of 28 weeks in which participants returned to a normal diet and were trained for weight maintenance. After this 12-month intervention, patients were further followed up for another 12 months. During the two-year-period, participants underwent a detailed medical examination at baseline and every six months. All individuals collected stool samples in stool collection tubes containing DNA/RNA stabilizer (lnvitek Molecular GmbH, Berlin, Germany; ref: 10381I1300) at the day of the study visits at home. After collection, the stool tubes were kept at −20 °C in thermal packs and transported to the Metabolic Unit where they were stored at −80 °C. All fecal samples relevant for the present study were collected in 2010 and 2011. Metagenomic sequencing was performed for all samples in one run in 2015. For the present analysis, the baseline microbiota patterns were analyzed in relation to the outcome 24 months later. Outcome variables were RWL and change in BMI (delta BMI) from baseline to month 24.

### 2.2. Analysis of Gut Microbiota

For whole metagenome analysis, we used shotgun sequencing of stool DNA to assess taxonomic and functional features at baseline, as described earlier in detail [7]. DNA was sequenced on an Illumina HiSeq 2500 Sequencer by °CeGat Inc., Tübingen, Germany. Samples (50 ng as quantified by Qbit) were processed with the Illumina ‘Nextera-DNA-Sample-Preparation Kit’ according to manufacturer’s protocol. Sequencing was performed with 2 × 100 nucleotides (paired-end sequencing) on 8 lanes with 300 GB raw data. On average, the sequencing achieved 2.1 GB/sample. Samples were sequenced with a sequencing depth of 10.9 million reads per paired-end sequencing file (s = 6.3 million). Raw sequences obtained from 15 metagenomic samples (15 patients, only baseline data) were subjected to a quality check using the FastQC software (www.bioinformatics.babraham.ac.uk/projects/fastqc/; accessed on 6 November 2021). Quality check comprised per base sequence quality, per-sequence quality scores, per-base sequence content, per-sequence GC content, per-base N content, sequence length distribution, sequence duplication levels, kmer content, and over-represented sequences. All samples showed satisfactory values for each parameter tested. Next, the sequences were processed using PRINSEQ for removing low-quality reads, trimming of poly-Ns, and A/T tails [10]. Each sample was subjected to a BLASTX analysis against the NCBI-NR database using an in-house developed tool (MALT, http://ab.inf.uni-tuebingen.de/software/malt/; accessed on 4 November 2021) with a maximum allowed e-value of 1.0. The BLASTX files were imported into MEGAN5 (http://ab.inf.uni-tuebingen.de/software/megan5/; accessed on 6 November 2021). MEGAN5 carries out binning of the reads into taxonomic and functional categories based on the BLASTX hits. The minimum bit score used for the analysis was 50 and a minimum support of 50 reads for each taxonomic category was used for the LCA algorithm. Ultimately, reads were assigned to a taxonomic and functional category. On average, about 50% of the reads in each sample were assigned to some category, 79% thereof were down to the level of genera and about 61% to the level of species. The samples were normalized with respect to each other. The functional annotation of the reads was performed based on the KEGG library (Kyoto Encyclopedia for Genes and Genomes, http://www.genome.jp/kegg/; accessed on 6 November 2021). Microbiome data handling was in line with recent recommendations [11]. Metagenomic data are available in the NCBI database under Bioproject ID PRJNA290729.

### 2.3. Correlation Coefficients

Correlation coefficients between each predictor variable from the set of genera/species on one hand and delta BMI/RWL on the other hand were calculated and ranked to obtain a first overview of potentially promising candidate variables (Supplementary Table S1).

### 2.4. Metagenomic Data Sets Used for Statistical Modeling

Determination of the gut microbiome of the patients led to two native data sets comprising the bacterial genera and the bacterial species data. Both these data sets contained the patient ID, the success rate (PS or NS), RWL and delta BMI, and relative abundances at baseline for the respective genera and species, i.e., the relative abundances of 1020 different bacterial genera (first data set) and 2529 different bacterial species (second data set). Next, the data sets were reduced to contain only the first 102 (first data set, genera) and 106 (second data set, species) columns, corresponding to the most abundant genera or species, which cover ~99% (first data set, genera), and ~96% (second data set, species) of the gut microbiome, respectively. The rationale of this approach is due to many genera or species only occurring in a low number of individuals, and at very low frequencies; thus, increasing the potential of a considerable effect of measurement errors. In case of the genera data set, three genera (*Caldicellulosiruptor*, *Thermaerobacter,* and *Thermobacillus*) were eliminated prior to reduction, as they do not normally occur in the gut microbiome, and hence, are likely misclassifications. Third, the respective proportions of genera and species were calculated and represented the values of the predictor variables for each data set.

The definition of regression models and the subsequent model selection both require a set of suitable terms (*T = {x_i_, i* = 1, …, *p*}). In our case, the number of predictor variables (*p* = 102 and *p* = 106 for the two data sets) is quite large compared to the number of patients (*n* = 15). Therefore, three avenues were pursued to locate the most important predictor variables contained in *T*: (I) the determination of the correlation between each predictor variable and delta BMI/RWL, (II) an elastic net regularization approach, and (III) multiple linear regression models in a Monte Carlo approach to complement the set of the most important predictor variables (for details see Supplementary Table S3A,B).

### 2.5. Ordination and Differential Abundance Analyses

Prior to the downstream analyses, microbial raw count data were converted into different forms: relative abundance, Z score of relative abundance, and centered log-ratio transformed (Clr) data. Clr data were used in principal component analyses (PCA) and in computing the Euclidean distance for permutational multivariate analysis of variance (PERMANOVA) tests using the Adonis function in the vegan package [12] (further details are described in [13]). While bacterial abundance was visualized by Z scores, Clr data of individual taxa between the PS and NS groups were compared using Mann–Whitney U tests with the respective *p*-values being adjusted with a false discovery rate of 5%. All analyses were performed using R version 4.1.2 (R Core Team: www.r-project.org, Vienna, Austria; accessed on 23 November 2021).

## 3. Results

### 3.1. Elastic Net Regularization

As the number of predictor variables *p* is high compared to the sample size n, elastic net regularization, a regularized regression method, was subsequently applied. This results in a model with certain terms that are present or absent for each repeat and each setting (defined by the number of folds and value of the elastic net mixing parameter α), always for the optimal λ cross-validated in the same repeat. As responses that quantify the success of the intervention, both delta BMI and RWL were used independently. Applying this method, we identified four bacterial genera and three bacterial species as candidates for predicting success of the weight-loss intervention: the genera were *Akkermansia* (class *Verrucomicrobia*), *Alistipes* (class *Bacteroidia*), *Symbiobacterium,* and *Pseudoflavonifractor* (both class *Clostridia*), while the species were *Alistipes finegoldii*, *Akkermansia muciniphila,* and *Ethanoligenens harbinense* (Table 2 and Supplementary Table S2).

Only predictor variables with an occurrence of at least 1% over all repeats for one of the response variables (delta BMI or RWL) are shown. Variables have been sorted by highest values for genera and species. In both cases, ∝ = 1 and 5 folds were used. Genera and species that were eventually selected are in boldface. The same information for other values of ∝, and 3, 8, or 15 folds, is shown for both genera and species in Supplementary Table S2.

### 3.2. Monte Carlo (MC) Approach Using Multiple Linear Regression Models

After the elastic net regularization model suggested an initial set of predictor variables, this set was complemented using an MC approach that tested a large number of multiple linear regression models. Table 3 and Table 4 (and Supplementary Table S3, which in addition contains the detailed calculation) show the most useful predictor variables for the genera and species data set and two different weightings. The numbers in both tables represent the weighted indexes as described in the methods section, with higher values indicating more-important terms.

### 3.3. Final List of Selected Predictor Variables

The predictor variables selected by elastic net regularization and the MC approach were combined to a set of terms that represent the most-promising candidates to predict weight loss according to the present data set. Table 5 pools and summarizes the results of both selection methodologies, listing seven genera and eight species that should be considered for weight-loss prediction.

### 3.4. Weight-Loss Prediction

Using predictor variables from Table 5, linear regression models with 1–5 terms were created from the set of variables and fitted (i.e., the coefficients *β_j_* were calculated) for both genera and species, and for delta BMI and RWL. The best models for each number of terms and their predictions for each patient, when applied to the data sets with an added weight-loss column, are listed in Table 6 and—in more detail—in Supplementary Tables S4 (models and model verification via cross-validation) and S5 (weight-loss predictions; relative and absolute deviations of the predicted weight loss to the observed weight loss).

In case of the genera, a model with 4 terms (*df* = 6) had the lowest AICc in terms of the delta BMI response (74.9), while the best models for 3 and 4 terms had nearly equal AICc values (100.3 vs. 101.2) for the RWL response. The terms used in these models were *Marvinbryantia*, *Megasphaera*, and *Symbiobacterium* in the case of the model with just three terms and the RWL response, and *Marvinbryantia*, *Megasphaera*, *Symbiobacterium,* and *Blautia* in the other cases. For both responses, relative standard errors for the coefficients varied between 12% and 50%, while adjusted R^2^ value varied between 82% and 86%. Estimating an R^2^ value for an independent test data set by performing a cross-validation on the data set with 3, 5, 8, and 15 folds, and calculating the R^2^ value from the averaged predictions, we obtained values between 67% and 69%, again for both responses.

For the species, a model with four terms (*df* = 6) had the lowest AICc in all cases, and the terms used in the models were *Alistipes finegoldii*, *Bacteroides caccae*, *Bacteroides stercoris,* and *Roseburia intestinalis* in all cases. The relative standard errors for the coefficients varied between 10% and 31%, the adjusted R^2^ value ranged between 91% (delta BMI) and 93% (RWL), and the cross-validated R^2^ value from the averaged predictions were ~80% for the delta BMI response and 83–85% for the RWL response, depending on the number of folds used (see Supplementary Table S4).

**Figure 1:**
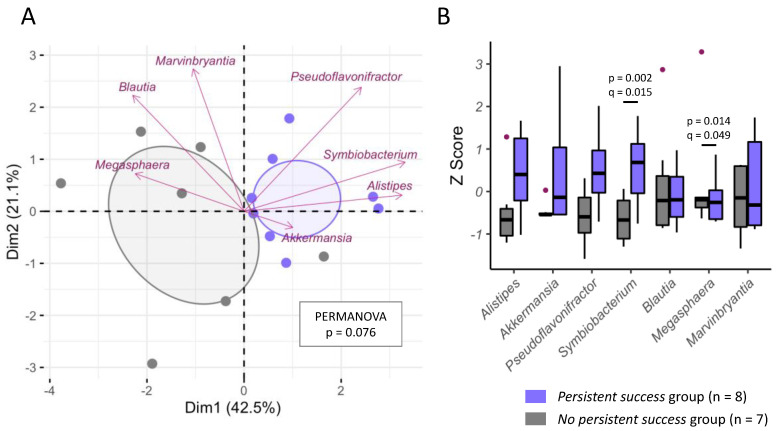
Microbial differences between individuals from the persistent success and the nonpersistent success groups on genus level. (**A**) This principal component analysis plot shows the 7 weight-loss predictive genera as well as the 15 individuals, color-coded by their persistent/nonpersistent weight loss status. Distances between the dots (representing individuals) were computed by the Euclidean index on centered-log ratio transformed data of the seven weight-loss predictive genera presented in Table 5. Dim, dimension/principal component; PERMANOVA, permutational multivariate analysis of variance. (**B**) Boxplots showing the comparisons of bacterial abundance (Z score) between the two groups for each genus using Mann–Whitney U tests with the respective *p*-values being adjusted with the false discovery rate method (q-value). Only comparisons with q < 0.1 are shown.

**Figure 2:**
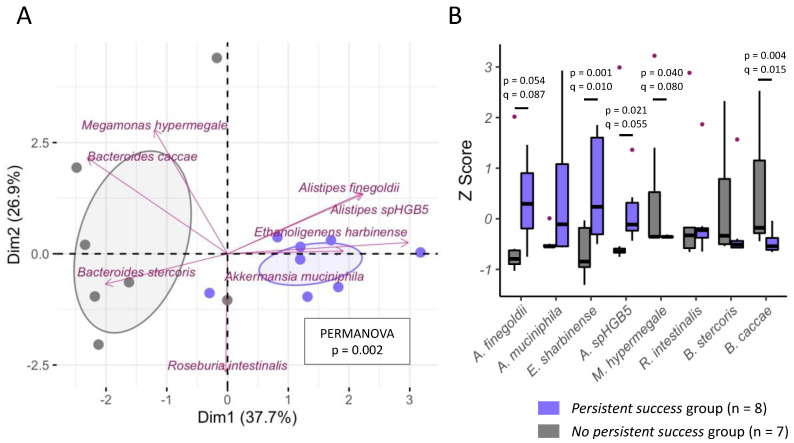
Microbial differences between individuals from the persistent success and the nonpersistent success groups on species level. (**A**) This principal component analysis plot shows the 8 weight-loss predictive species as well as the 15 individuals, color-coded by their persistent/nonpersistent weight-loss status. Distances between the dots (representing individuals) were computed by the Euclidean index on centered-log ratio transformed data of the eight weight-loss predictive species shown in Table 5. Dim, dimension/principal component; PERMANOVA, permutational multivariate analysis of variance. (**B**) Boxplots showing the comparisons of bacterial abundance (Z score) between the two groups for each species using Mann–Whitney U tests with the respective *p*-values being adjusted with the false discovery rate method (q-value). Only comparisons with q < 0.1 are shown.

Finally, to illustrate the findings of our prediction models, we plotted the predictive genera and species from Table 5 using PCA. As shown in Figure 1A, there was a trend of different genera patterns between the PS and the NS groups in terms of the seven predictive genera (PERMANOVA, *p* = 0.076). In detail, patients who were successful in the program had a higher abundancy of *Akkermansia*, *Alistipes*, *Pseudoflavonifractor*, and *Symbiobacterium* genera (Figure 1B). On the species level, there was a clear separation between successful and nonsuccessful individuals in terms of the eight predictive species (PERMANOVA, *p* = 0.002, Figure 2A). Most dominantly, successful individuals had a higher abundancy of *Alistipes finegoldii* and *Ethanoligenens harbinense*, whereas nonsuccessful individuals had a higher abundancy of *Bacteroides caccae* (Figure 2B).

## 4. Discussion

Prediction variables for weight loss are of major interest for the care of patients with obesity. It is known that the type of intervention and individual factors influence the outcome of weight-reductions means [2,3]. Among the individual factors, the intestinal microbiota is a highly interesting data source that might yield such predictive variables, since it has been shown that obesity and metabolic processes and disease are affected by individual microbiota patterns [6,7,8,13,14,15]. Here, we show for the first time that defined microbiota patterns can be related to individual weight loss success following a nonsurgical, multidisciplinary weight loss program performed over one year. Baseline gut microbiota analysis allowed the identification of bacterial patterns that might predict long-term weight loss success two years after start of the multidisciplinary weight loss program. The results provide information on the relative importance of predictors at the genus and species level. On the genus level, relative abundance of *Symbiobacterium* was the most important predictor variable in the present study, as it was selected in every approach applied, followed by *Megasphaera*, which showed a very strong performance in the MC approach, and a moderately negative correlation to delta BMI and RWL. On the species level, relative abundance of *Alistipes finegoldii* was identified as the most important predictor variable, being selected by every approach while showing a substantial positive correlation with delta BMI and RWL.

Using the seven genera or the eight species selected by the MC procedure in the present analyses, linear regression models were fitted. Comparing all models as before using the AICc, the predictive power of the best model for each *df* was determined. On the one hand, finding models to make accurate predictions regarding weight loss using the proportions of genera or species of gut microbes of a patient is rather unrealistic given the low sample size in the present study. On the other hand, it is nevertheless helpful to calculate summary statistics of models containing only genera and species from the final lists of the MC procedure in order to identify the most important terms of the final set of terms. This also provides a rough estimate of standard statistics of models composed only of predictor variables from the final set, such as R^2^ values and relative standard errors of the estimated coefficients of the terms. The adjusted R^2^ values, but also the cross-validated R^2^ values, appear to be higher than what could be expected, considering the small sample size. This is in part owing to the methodology of the present analyses, as the large number of predictor variables considered in combination with the MC approach applied some correlations between explanatory variables and the response might be the result of chance rather than represent a genuine relationship. While we are aware of the risk that not all 15 identified genera and species might be useful predictors, we nevertheless consider our approach to be a useful strategy to determine candidates for predictor variables when the sample size is low but the number of predictors is relatively high.

The predicted weight loss success for each patient in the present study, based on the optimal model for a specified number of terms, revealed a high rate of successful predictions for all models with at least 2 terms, and also for the models with genera as predictors, even though these models have, on average, lower predictive power than those with species as predictors. However, as large numbers of linear regression models were fitted with the MC method, a certain amount of overfitting might be expected, even as we tried to counter that by using the AICc, and the results should be validated with a larger cohort of patients.

Our present analysis revealed a set of seven bacterial genera and eight species from the commensal intestinal microbiota that are potential candidates to predict the likelihood of long-term weight loss success. In our previous studies, the genera *Alistipes*, *Pseudoflavonifractor*, and *Symbiobacterium* were shown to be significantly more abundant in the PS group [7]. At the species level, several *Bacteroides* species were less abundant, while *Clostridium leptum* was more abundant in the PS group compared to the NS group [7]. In the present study we confirm by using predictive modeling that the genera *Symbiobacterium*, *Alistipes,* and *Pseudoflavonifractor,* and the species *Bacteroides caccae* and *Bacteroides stercoris,* are candidate indicators for persistent success following the weight-loss intervention program. Moreover, we identified an additional four genera (*Megasphaera*, *Marvinbryantia*, *Blautia*, and *Akkermansia*) and six species (*Alistipes finegoldii*, *Alistipes spHGB5*, *Roseburia intestinalis*, *Akkermansia muciniphila*, *Ethanoligenens harbinense*, and *Megamonas hypermegale*) as candidate indicators for persistent success in weight loss. Preliminary confirmation studies revealed that a single genera or a single species of bacteria are obviously insufficient to make a prediction (own unpublished results); however, it is likely that the identified patterns of bacterial genera or species do allow a prediction of weight loss success. The present study constitutes an important first step to assess possible associations between gut microbial composition and success of a multidisciplinary weight loss program. Based on the findings shown here, future studies can be designed to establish specific recommendations and guidelines for clinical practice, e.g., thresholds for each of the predictive bacteria.

Our findings on weight-loss prediction by microbial patterns based on statistical analyses are confirmed by biological considerations regarding the known functionality of the bacterial candidates we identified [16]. For example, several studies have demonstrated an inverse association between obesity and the abundance of *Alistipes* in the gut. *Alistipes* was more abundant at baseline in PS participants. This is in line with Lapthorne et al. [17], who showed that microbiota changes associated with surgery included a decreased relative abundance of *Alistipes*. In a recent study, the abundance of *Alistipes* was positively correlated with body weight, fat mass, serum cholesterol and triglycerides, leptin, IL-6 and lipopolysaccharide contents, as well as PPARγ gene expression in mice [18].

According to our data, *Akkermansia* is another potential indicator for persistent weight loss success. Individuals with higher abundance of *A. muciniphila* at baseline have been shown to display a greater improvement in insulin sensitivity markers and body composition after dietary intervention [19]. In humans, studies have provided evidence for a negative correlation between *A. muciniphila* abundance and being overweight, obesity, untreated type 2 diabetes mellitus, or hypertension [20,21,22,23,24]. Our results are in line with this, since patients had a lower mean BMI and a higher abundance of *A. muciniphila* at the end of the weight-loss intervention program than at baseline. In another study, it was shown that relative abundances of several species including those of *Alistipes* spp. and *A. muciniphila* increased after Roux-en-Y gastric bypass surgery in parallel with weight loss and metabolic improvements [25].

Of interest are also the genera of *Symbiobacterium* and *Pseudoflavonifractor*, which were more abundant in the PS group of patients, both in our previous [7] and present study. *Symbiobacterium* comprises four species, of which *S. thermophilum*, a syntrophic bacterium that lives in strict symbiosis with *Bacilli*, has been studied most extensively [26]. However, so far none of the *Symbiobacterium* species had been related to obesity or metabolic disease. The genus *Pseudoflavonifractor* only consists of two species, which have also never been associated with obesity or metabolic disease. Thus, these groups of bacteria warrant more attention, especially in the context of obesity-related diseases.

In addition, the genus *Megasphaera* was found elevated at baseline in the PS group and thus can be considered to be a possible predictor for weight loss success. A major bacterial population found that present or elevated in postsurgery subjects with obesity is related to the species *M. elsdenii* [27]. It has been shown that the fecal microbiota of postsurgery patients was significantly enriched in *Megasphaera* [28]. *Megasphaera* abundance is associated with blood glucose and insulin levels [29], as well as *Blautia*, implicating a role for these two genera in host glucose metabolism [30]. The genera *Marvinbryantia* and *Blautia* that we newly identified as possible predictors for weight loss have been positively correlated with body weight [31].

Among the bacteria associated with lower risk of weight gain, several operational taxonomic units (OTUs) assigned to the family of *Ruminococcacaeae* were found [32,33,34]. In line with this, the genus *Oscillibacter* belonging to this family was significantly under-represented in patients with nonalcoholic fatty liver disease (NAFLD) [35]. Other studies have also associated the abundance of *Oscillibacter* with obesity [30,36,37]. In our study, the abundance of *Oscillibacter* was a determinant of weight loss success, although less prominent than the genera discussed before.

On the species level, eight bacterial species were identified which might have a major impact on weight-loss prediction. Apart from *A. finegoldii*, *A. spHGB5,* and *A. muciniphila*, several butyrate-producing species were identified by our statistical approach, such as *Roseburia intestinalis*, which was selected as a predictor variable for weight loss success from the MC approach using multiple linear regression models. Consistent with our findings, results from animal studies have shown that an increased abundance of *R. intestinalis* was associated with weight loss and reduced glucose intolerance [38]. In humans *R. intestinalis* was shown to be present at lower concentrations in individuals with type 2 diabetes compared to healthy subjects [39,40,41,42]. Similarly, *Bacteroides stercoris* (and likely also *Bacteroides caccae*) have been identified as butyrate producers [43].

In our previous study [7], the bacterial genera *Megamonas* and *Prevotella* were identified as strong markers in the NS group. In the present analysis, *Megamonas hypermegale* was also identified as a predictor variable for weight loss success from the MC approach. To our knowledge, these are the first data in humans hinting at a link of *Megamonas* with body weight. Consistent with our results, *Megamonas* abundance was found to be negatively correlated with weight-loss rate in dogs [44]. *Prevotella dentalis* was also found as a predictor variable using the elastic net regularization, though it was not among the top predictors. In contrast, *Ethanoligenens harbinense*, on our list of predictors, has not been studied, to our knowledge, in the context of obesity so far.

Our study has both strengths and limitations that deserve consideration. The rather small sample size of 15 individuals may limit the explanatory power of our analyses. However, the accuracy of our prediction model is high; therefore, the power should be, in fact, sufficient, despite the small number of participants. The strengths of our study comprise the high quality of microbiome sequencing by shotgun sequencing, and the up-to-date multivariate statistical approach. Nevertheless, our findings need confirmation by larger prospective trials.

## 5. Conclusions

We identified seven genera and eight species within the fecal commensals which might predict persistent weight loss success in obese individuals undergoing nonsurgical therapy. Assessment of these commensals by next generation sequencing or routine qPCR techniques could allow personalized recommendations regarding whether an individual should follow the nonsurgical weight-loss approach or not, increasing the success rate in obesity therapy. Despite a relatively small sample size, the detailed and complex statistical analysis enabled an astonishingly accurate prediction of success and failure with regards to long-term weight loss. These findings warrant further analyses in future confirmatory studies.

## Figures and Tables

**Table 1:** Characteristics of the study population.

Parameter	Hypothesis-Generating Cohort
*n*	15
Weight loss success (*n*)	8
Age	40 ± 6
Blood pressure systolic (mmHg)	126 ± 15
Blood pressure diastolic (mmHg)	86 ± 11
Fasting blood glucose (mg/dL)	105 ± 619
Cholesterol (mg/dL)	203 ± 641
HDL cholesterol (mg/dL)	47 ± 610
LDL cholesterol (mg/dL)	130 ± 27
Triglycerides (mg/dL)	181 ± 154
WC (cm)	123 ± 15
Weight (kg)	128 ± 20
BMI (kg/m^2^)	42.4 ± 6

Weight loss success is defined as relative weight loss at T24 > 10%. Data are presented as mean ± standard deviation. Abbreviations: T24 = 24 months after baseline, HDL = high-density lipoprotein, LDL = low-density lipoprotein, WC = waist circumference, BMI = body mass index.

**Table 2:** Selection of genera and species predictor variables according to elastic net regularization.

Predictor Variable		Occurrence (Delta BMI)	Occurrence (RWL)
Genera	*Akkermansia*	10.40%	46.10%
	*Symbiobacterium*	3.40%	51.80%
	*Alistipes*	10.40%	26.40%
	*Pseudoflavonifractor*	6.90%	15.90%
Species	*Alistipes finegoldii*	100.00%	22.00%
	*Akkermansia muciniphila*	10.10%	19.10%
	*Ethanoligenens harbinense*	0.30%	17.10%
	*Bacteroides ovatus*	0.20%	1.30%
	*Bacteroides eggerthii*	0.00%	1.00%

**Table 3:** Selection of genera predictor variables using the Monte Carlo approach.

Genera (Weight 1)	Full List	Reduced List	Reduced List	Reduced List	Reduced List
(A 60%, B 20%, C 20%)	(102 Terms)	(44 Terms)	(23 Terms)	(15 Terms)	(7 Terms)
**Megasphaera**	0.9957	0.9570	0.9515	0.9397	0.9551
**Symbiobacterium**	0.8742	0.7755	0.6916	0.6964	1.0000
**Marvinbryantia**	0.7179	0.7126	0.7805	0.8054	0.7513
**Blautia**	0.5285	0.5802	0.5475	0.5781	0.6515
**Dysgonomonas**	0.3212	0.6202	0.7111	0.7311	0.3895
Oscillibacter *	0.3818	0.5506	0.5359	0.5225	0.5057
**Pseudoflavonifractor**	0.4032	0.4131	0.4091		0.4474
Burkholderia	0.3751	0.5381	0.5932	0.6016	
Treponema	0.3780	0.5865	0.6172	0.6445	
Aeromonas	0.3029	0.4871	0.5573	0.5955	
Gordonibacter	0.3518	0.3991			
Streptococcus	0.3041	0.4350	0.4969	0.5200	
Alistipes	0.5047	0.4468	0.4249		
Haemophilus	0.3812	0.4531	0.4335		
Bordetella	0.3540	0.4177	0.4631	0.4894	
**Genera (weight 2)**	**Full List**	**Reduced List**	**Reduced List**	**Reduced List**	**Reduced List**
**(A 45%, B 10%, C 45%)**	**(102 Terms)**	**(52 Terms)**	**(25 Terms)**	**(15 Terms)**	**(7 Terms)**
**Megasphaera**	0.9973	0.9981	0.9686	0.9936	0.9609
**Symbiobacterium**	0.9162	0.8792	0.8201	0.8379	1.0000
**Marvinbryantia**	0.6671	0.6583	0.7758	0.7302	0.7180
**Blautia**	0.6315	0.6632	0.6414	0.6044	0.6844
**Dysgonomonas**	0.3434	0.5460	0.7008	0.7817	0.4112
Oscillibacter *	0.4706	0.5025	0.5379	0.5652	0.5645
**Pseudoflavonifractor**	0.4946	0.4854	0.4529		0.5026
Burkholderia	0.4192	0.5271	0.6486	0.6774	
Treponema	0.4055	0.4918	0.5667	0.5731	
Aeromonas	0.3337	0.4694	0.5539	0.6356	
Gordonibacter	0.4607	0.4597			
Streptococcus	0.3485	0.5570	0.5711	0.6459	
Alistipes	0.6135	0.5296	0.4784		
Haemophilus	0.5137	0.5667	0.5167	0.5037	
Bordetella	0.3599	0.4661	0.5346	0.6305	

Predictor variables for the genera data set and weightings 1 and 2 are shown in the table. Genera that were eventually selected are in boldface. Italics represent genera that were already selected in the previous step (elastic net regularization). * Oscillibacter was selected as it was one of the top predictor variables (together with Blautia) in a reduced data set that only tested the 46 most common genera (data not shown). Detailed results including all 102 genera, as well as the calculation of the index values for the full list (102 terms), and weighting 2 can be found in Supplementary Table S3.

**Table 4:** Selection of species predictor variables using the Monte Carlo approach.

Species (Weight 1)	Full List	Reduced List	Reduced List	Reduced List	
(A: 60%, B: 20%, C: 20%)	(106 Terms)	(28 Terms)	(16 Terms)	(8 Terms)	
**Alistipes finegoldii**	0.9043	0.6981	0.7663	0.9521	
**Roseburia intestinalis**	0.6873	0.9467	0.9814	0.8594	
**Alistipes spHGB5**	0.6824	0.8763	0.9004	0.6096	
**Bacteroides caccae**	0.6871	0.6270	0.6522	0.8361	
**Megamonas hypermegale**	0.5777	0.7872	0.8143	0.6423	
**Bacteroides stercoris**	0.4309	0.7694	0.8154	0.6728	
**Ethanoligenens harbinense**	0.3782	0.4657	0.4623	0.4312	
**Akkermansia muciniphila**	0.4986	0.4376	0.4079	0.4461	
Prevotella dentalis	0.3424	0.5949	0.6055		
Bifidobacterium bifidum	0.4589	0.5286	0.5876		
Pseudoflavonifractor capillosus	0.3900	0.5629	0.5214		
Bacteroides ovatus	0.3736	0.5577	0.5795		
Clostridium hathewayi	0.3342				
**Species (Weight 2)**	**Full List**	**Reduced List**	**Reduced List**	**Reduced List**	**Reduced List**
**(A: 45%, B: 10%, C: 45%)**	**(106 Terms)**	**(32 Terms)**	**(21 Terms)**	**(15 Terms)**	**(8 Terms)**
**Alistipes finegoldii**	0.9522	0.8361	0.8729	0.8527	0.9760
**Roseburia intestinalis**	0.6167	0.8676	0.9245	0.9609	0.8501
**Alistipes spHGB5**	0.6871	0.8249	0.8489	0.8990	0.6666
**Bacteroides caccae**	0.7308	0.7343	0.7362	0.7914	0.8713
**Megamonas hypermegale**	0.5581	0.7228	0.7384	0.7799	0.7011
**Bacteroides stercoris**	0.4554	0.6997	0.7370	0.7894	0.6564
**Ethanoligenens harbinense**	0.5017	0.5354	0.5162	0.5548	0.4834
**Akkermansia muciniphila**	0.6040	0.5443	0.5195	0.4576	0.5029
Prevotella dentalis	0.3577	0.6112	0.6224	0.6351	
Bifidobacterium bifidum	0.4012	0.5426	0.5808	0.6033	
Pseudoflavonifractor capillosus	0.5032	0.6374	0.5877	0.5341	
Bacteroides ovatus	0.3828	0.5227	0.5328	0.5515	
Clostridium hathewayi	0.2808				

Predictor variables for the species data set and weightings 1 and 2 are shown in the table. Species that were eventually selected are in boldface. Italics represent genera that were already selected in the previous step (elastic net regularization). Detailed results including all 106 species, as well as the calculation of the index values for the full list (106 terms), and weighting 2 can be found in Supplementary Table S3.

**Table 5:** Most-relevant candidates on the genera and species level for weight loss success predictor variables.

	Weight (Importance)				
	1	1	3	1	
**Selected genera**	**Full MC list** **(102 terms)** **Average *rank***	**Final MC list** **(7 terms)** **Average *rank***	**Elastic net** **selected**	**Strong** **Correlation** **(+ or −)**	**Final score**
*Symbiobacterium*	2	1	1	1.5	15.5
*Megasphaera*	1	2	-	–0.5	11.5
*Marvinbryantia*	3	3	-	-	8
*Alistipes*	5	-	1	1.5	6.5
*Blautia*	4	4	-	-	6
*Akkermansia*	-	-	1	1.5	4.5
*Pseudoflavonifractor*	-	-	1	0.5	3.5
**Selected species**	**Full MC list (106 terms)** **Average *rank***	**Final MC list (8 terms)** **Average *rank***	**Elastic net** **selected**	**Strong** **Correlation** **(+ or −)**	**Final score**
*Alistipes finegoldii*	1	1	1	2	17
*Bacteroides caccae*	3	2.5	-	−0.5	9
*Roseburia intestinalis*	3	2.5	-	-	8.5
*Akkermansia muciniphila*	5	-	1	1.5	6.5
*Alistipes* spHGB5	3	5.5	-	-	5.5
*Megamonas hypermegale*	5	4.5	-	-	4.5
*Ethanoligenens harbinense*	-	-	1	1	4
*Bacteroides stercoris*	-	5	-	-	2

Final sets of selected genera and species are shown in the table. A final score was calculated, based on a set of weights (top line) for the different approaches to identify predictor variables. The first column of the table contains the names of the selected genera and species; the next two columns show the ranks of the predictor variables for the full (second column) and final (third column) lists in the Monte Carlo (MC) approach. The fourth column shows the predictor variables selected by the elastic net approach (1), while the fifth column identifies predictor variables with strong correlations (positive or negative) to both delta BMI and RWL. Negative correlations are identified with a minus sign, and the absolute values are calculated by grouping the predictor variables into four classes based on strength of correlation: for an absolute value of correlation between 45% and 50%, rounded to full percent, a value of 0.5 is assigned, between 50% and 55%, a value of 1 is assigned, between 55% and 60%, a value of 1.5 is assigned, and 60% and above leads to a value of 2. The final score for each predictor variable is calculated by subtracting the MC ranks (columns 2 and 3) from 7 and multiplying them with the respective weights (1 in both cases) before summing up these two values with the product of the value of column 4 with its weight (3) and the absolute value of column 5 with its weight (1). Only predictor variables with final scores of 2 and above are shown.

**Table 6:** Classification of relative weight-loss predictions for each patient under different models.

	Classification	Classification	Classification	Classification	Classification
	RWL	RWL	RWL	RWL	RWL
DS	1 Term Model	2 Terms Model	3 Terms Model	4 Terms Model	5 Terms Model
1	correct(−)	correct(−)	correct(−)	correct(−)	correct(−)
2	correct(−)	correct(−)	correct(−)	correct(−)	correct(−)
3	**underpredicted**	**underpredicted**	correct(+)	correct(+)	correct(+)
4	correct(−)	correct(−)	correct(−)	correct(−)	correct(−)
5	correct(−)	correct(−)	correct(−)	correct(−)	correct(−)
6	correct(+)	correct(+)	correct(+)	correct(+)	correct(+)
7	correct(−)	correct(−)	correct(−)	**overpredicted**	correct(−)
8	correct(+)	correct(+)	correct(+)	correct(+)	correct(+)
9	correct(+)	correct(+)	correct(+)	correct(+)	correct(+)
10	**underpredicted**	correct(+)	correct(+)	correct(+)	correct(+)
11	**overpredicted**	correct(−)	correct(−)	correct(−)	correct(−)
12	**underpredicted**	correct(+)	correct(+)	**underpredicted**	**underpredicted**
13	correct(−)	correct(−)	correct(−)	correct(−)	correct(−)
14	**underpredicted**	**underpredicted**	**underpredicted**	correct(+)	correct(+)
15	correct(+)	correct(+)	correct(+)	correct(+)	correct(+)
**correct(+)**	4	6	7	7	7
**correct(−)**	6	7	7	6	7
**underpredicted**	4	2	1	1	1
**overpredicted**	1	0	0	1	0
**sum correct**	**10**	**13**	**14**	**13**	**14**
**sum incorrect**	**5**	**2**	**1**	**2**	**1**

Classification of the predicted relative weight loss (RWL) for each patient under the optimal linear regression models with one to five linear terms containing predictor variables from the final set of selected species of Table 5 is shown. Predictions for patients, numbered from 1–15 (column “DS”) were classified as “correct(+)” in cases where significant weight loss occurred and was predicted, as “correct(−)” in cases where significant weight loss did not occur and was predicted as not occurring, as “overpredicted” in cases where a model predicted significant weight loss but it did not occur, and as “underpredicted” in cases where a model predicted no significant weight loss but significant weight loss occurred. The threshold for a significant relative weight loss was chosen as 10. Analogous tables for delta BMI, and for predictor variables from the final set of selected genera of Table 5 for both RWL and delta BMI, can be found in Supplementary Tables S5 and S6.

## Data Availability

Metagenomic data are available in the NCBI database under Bioproject ID PRJNA290729. All data described in the manuscript, code book, and analytic code will be made available upon request pending approval by the corresponding author S.C.B.

## Supplementary Materials

The following supporting information can be downloaded at: https://www.mdpi.com/article/10.3390/nu14153182/s1, Supplementary Tables S1–S6. Table S1: Correlations between genera/species and both delta BMI and RWL. Table S2: Elastic net regularization. Table S3: Calculation of importance indices and ranks and a combined index value. Table S4: Linear regression models. Table S5: Predicted weight loss, calculated as delta BMI. Table S6: Predicted weight loss, calculated as relative weight loss.
