# Supplementary material for: Gut Microbiota Patterns Predicting Long-Term Weight Loss Success in Individuals with Obesity Undergoing Nonsurgical Therapy
Source: Nutrients. 2022 Aug 3;14(15):3182. doi: 10.3390/nu14153182 (PMC9370776; doi:10.3390/nu14153182)
Supplement: Supplementary file 1 [file nutrients-14-03182-s001.zip › nutrients-1830543-supplementary.pdf]

Supplementary Tables S1-S6

Supplement to: Gut microbiota patterns predicting long-term weight loss success in individuals with obesity undergoing non-surgical therapy

Stephan C. Bischoff[1*], Nguyen K. Nguyen[2], Benjamin Seethaler[1], Julia Beisner[1], Philipp Kügler[3], Thorsten Stefan[3]

[1]Institute of Nutritional Medicine, University of Hohenheim, Stuttgart

[2]Microbiome Insights Inc, Vancouver, Canada

[3]Institute of Applied Mathematics and Statistics & Computational Science Lab, University of Hohenheim, Stuttgart Corresponding author:

Stephan C. Bischoff, M.D., Professor of Medicine and Clinical Nutrition, Department of Nutritional Medicine, University of Hohenheim, 70593 Stuttgart, Germany, Phone +49 711 459 24101, Email bischoff.stephan@uni-hohenheim.de

Supplementary Table S1A

Correlations between each predictor variable from the set of genera and both delta BMI (column D) and RWL (column E). The mean correlation of both measures of weight loss can be found in column F. Scores were awarded for the strongest positive and negative correlations, with a mean correlation of r >= 60% corresponding to a score of 2, 55% <= r < 60% corresponding to a score of 1.5, 50% <= r < 55% corresponding to a score of 1, 45% <= r < 50% corresponding to a score of 0.5, and the same scores being awarded to strongly negative correlations accordingly.

Genus	Correlations (deltaBMI)	Correlations (RWL)	Correlations (mean)	Score
Alistipes	56,33%	57,90%	57,11%	1,5
Symbiobacterium	50,57%	58,81%	54,69%	1,5
Akkermansia	53,68%	55,59%	54,63%	1,5
Gordonibacter	48,14%	48,31%	48,22%	0,5
Ethanoligenens	42,78%	52,59%	47,69%	0,5
Eggerthella	47,23%	46,65%	46,94%	0,5
Pseudoflavonifractor	43,46%	48,71%	46,09%	0,5
Oscillibacter	39,43%	48,06%	43,75%	0
Mycobacterium	35,56%	45,92%	40,74%	0
Parasutterella	41,89%	39,38%	40,64%	0
unclassified_Ruminococcaceae	38,23%	41,76%	40,00%	0
Corynebacterium	34,81%	43,87%	39,34%	0
unclassified_Burkholderiales_miscellaneous	40,42%	36,78%	38,60%	0
Slackia	33,76%	41,99%	37,88%	0
Deinococcus	32,26%	43,46%	37,86%	0
Heliobacterium	31,45%	41,97%	36,71%	0
Fretibacterium	29,27%	39,07%	34,17%	0
Sphaerochaeta	29,73%	38,16%	33,95%	0
Treponema	28,82%	38,88%	33,85%	0
Streptomyces	28,25%	39,43%	33,84%	0
Enterococcus	29,19%	38,15%	33,67%	0
Desulfotomaculum	28,44%	37,72%	33,08%	0
Geobacter	27,32%	38,35%	32,84%	0
Subdoligranulum	30,82%	33,71%	32,27%	0
Anaeromyxobacter	26,03%	37,06%	31,54%	0
environmental_samples_Bacteria	28,53%	32,94%	30,74%	0
unclassified_sequences	24,30%	31,73%	28,02%	0
Paenibacillus	22,62%	32,93%	27,77%	0
Aeromonas	23,05%	31,46%	27,26%	0
Klebsiella	20,50%	33,76%	27,13%	0
Azospirillum	20,70%	32,67%	26,69%	0
Pseudomonas	21,47%	31,53%	26,50%	0
Geobacillus	20,32%	31,53%	25,93%	0
Anaerotruncus	22,19%	29,45%	25,82%	0
Syntrophobotulus	22,15%	28,73%	25,44%	0
Olsenella	22,47%	27,57%	25,02%	0
Desulfitobacterium	18,85%	28,14%	23,49%	0
Spirochaeta	17,94%	28,81%	23,38%	0
Rhizobium	16,63%	28,82%	22,73%	0
Selenomonas	17,22%	28,22%	22,72%	0
Escherichia	19,27%	25,89%	22,58%	0
Rhodospirillum	15,10%	26,78%	20,94%	0
Bradyrhizobium	14,65%	26,52%	20,59%	0
Bilophila	19,81%	20,97%	20,39%	0
Lactobacillus	12,69%	23,53%	18,11%	0
Dorea	20,83%	14,00%	17,41%	0
Odoribacter	13,76%	20,60%	17,18%	0
Butyrivibrio	14,56%	19,29%	16,93%	0
Ruminococcus	13,72%	19,44%	16,58%	0
Oribacterium	12,86%	19,35%	16,11%	0
Cellulosilyticum	11,65%	16,66%	14,15%	0
Propionibacterium	7,83%	19,52%	13,67%	0
Bacillus	8,14%	17,78%	12,96%	0
Coriobacterium	10,90%	13,89%	12,40%	0
Enterobacter	9,45%	14,34%	11,90%	0
Tannerella	12,29%	11,07%	11,68%	0
Dialister	5,40%	11,22%	8,31%	0
Acidaminococcus	9,81%	6,28%	8,04%	0
Staphylococcus	4,67%	11,10%	7,89%	0
Veillonella	6,64%	8,09%	7,37%	0
Bacteroides	8,56%	4,85%	6,71%	0
Coprococcus	2,09%	9,96%	6,03%	0
Barnesiella	3,50%	8,47%	5,99%	0
Flavobacterium	5,06%	6,49%	5,78%	0
Pedobacter	4,38%	6,61%	5,50%	0
Collinsella	5,22%	5,76%	5,49%	0
Desulfovibrio	1,79%	4,43%	3,11%	0
unclassified_Peptostreptococcaceae	0,27%	3,76%	2,02%	0
Faecalibacterium	-0,20%	3,90%	1,85%	0

Supplementary Table S1A (continued)

Marvinbryantia	-5,16%	3,92%	-0,62%	0
Clostridium	-4,15%	1,36%	-1,40%	0
Campylobacter	-5,12%	-0,73%	-2,93%	0
Paludibacter	-6,54%	-6,52%	-6,53%	0
Sutterella	-8,10%	-5,73%	-6,91%	0
unclassified_Lachnospiraceae	-8,91%	-5,83%	-7,37%	0
Capnocytophaga	-10,11%	-5,27%	-7,69%	0
Holdemania	-9,29%	-8,03%	-8,66%	0
Chitinophaga	-8,79%	-9,12%	-8,95%	0
Dysgonomonas	-13,57%	-5,80%	-9,68%	0
Prevotella	-8,60%	-12,66%	-10,63%	0
Sphingobacterium	-12,67%	-11,10%	-11,89%	0
unclassified_Erysipelotrichaceae	-15,46%	-9,09%	-12,27%	0
Paraprevotella	-15,48%	-10,20%	-12,84%	0
Cupriavidus	-15,78%	-10,99%	-13,38%	0
Bifidobacterium	-16,89%	-11,69%	-14,29%	0
Ralstonia	-16,99%	-12,65%	-14,82%	0
Acidovorax	-17,48%	-12,84%	-15,16%	0
Bordetella	-17,24%	-13,10%	-15,17%	0
Burkholderia	-20,09%	-15,00%	-17,55%	0
Bacteroides_pectinophilus	-18,00%	-17,29%	-17,64%	0
Streptococcus	-21,96%	-13,64%	-17,80%	0
Porphyromonas	-21,16%	-19,42%	-20,29%	0
unclassified_Clostridiales_miscellaneous	-21,23%	-20,27%	-20,75%	0
Phascolarctobacterium	-17,65%	-23,87%	-20,76%	0
Roseburia	-21,72%	-23,21%	-22,47%	0
Eubacterium	-22,83%	-25,21%	-24,02%	0
Megamonas	-23,95%	-27,74%	-25,84%	0
Parabacteroides	-34,42%	-31,37%	-32,89%	0
Human_endogenous_retroviruses	-37,95%	-32,05%	-35,00%	0
Blautia	-36,74%	-33,95%	-35,35%	0
Megasphaera	-47,61%	-43,79%	-45,70%	0,5
Haemophilus	-51,44%	-50,62%	-51,03%	1

Supplementary Table S1B

Correlations between each predictor variable from the set of species and both delta BMI (column D) and RWL (column E). The mean correlation of both measures of weight loss can be found in column F. Scores were awarded for the strongest positive and negative correlations, with a mean correlation of r >= 60% corresponding to a score of 2, 55% <= r < 60% corresponding to a score of 1.5, 50% <= r < 55% corresponding to a score of 1, 45% <= r < 50% corresponding to a score of 0.5, and the same scores being awarded to strongly negative correlations accordingly.

Species	Correlations (deltaBMI)	Correlations (RWL)	Correlations (mean)	Score
Alistipesfinegoldii	61,83%	58,81%	60,32%	2
Akkermansiamuciniphila	53,94%	55,88%	54,91%	1,5
Ethanoligenensharbinense	44,70%	54,68%	49,69%	1
Gordonibacterpamelaeae	49,49%	49,47%	49,48%	0,5
Pseudoflavonifractorcapillosus	45,27%	50,56%	47,92%	0,5
Bacteroidesintestinalis	49,28%	44,12%	46,70%	0,5
Oscillibactervalericigenes	41,10%	49,97%	45,53%	0,5
Alistipesputredinis	44,49%	44,40%	44,44%	0
Eggerthellalenta	42,19%	41,62%	41,90%	0
RuminococcaceaebacteriumD16	40,00%	43,28%	41,64%	0
Bacteroidescoprocola	43,33%	38,52%	40,93%	0
Ruminococcuschampanellensis	32,02%	43,20%	37,61%	0
Clostridiumleptum	34,83%	40,00%	37,41%	0
AlistipesspHGB5	38,76%	34,88%	36,82%	0
Subdoligranulumvariabile	32,71%	35,57%	34,14%	0
Eubacteriumlimosum	27,86%	36,62%	32,24%	0
unculturedorganism	25,91%	33,43%	29,67%	0
Bacteroidesovatus	29,05%	29,24%	29,15%	0
Anaerotruncuscolihominis	23,30%	30,68%	26,99%	0
Bacteroideseggerthii	25,26%	28,60%	26,93%	0
Dorealongicatena	28,79%	20,05%	24,42%	0
Eubacteriumeligens	24,29%	24,01%	24,15%	0
Alistipesshahii	21,03%	26,15%	23,59%	0
ClostridiumspSY8519	18,22%	26,86%	22,54%	0
Ruminococcusalbus	16,06%	28,86%	22,46%	0
Escherichiacoli	18,95%	25,19%	22,07%	0
Bacteroidescoprophilus	23,75%	19,70%	21,72%	0
Eubacteriumsiraeum	16,92%	26,35%	21,64%	0
Bacteroidessp3133FAA	22,28%	19,35%	20,82%	0
Butyrivibrioproteoclasticus	17,35%	24,13%	20,74%	0
Lachnospiraceaebacterium3157FAACT1	17,30%	22,43%	19,86%	0
Bifidobacteriumadolescentis	15,74%	22,61%	19,17%	0
Bilophilawadsworthia	18,09%	19,10%	18,59%	0
Bacteroidesclarus	19,37%	17,18%	18,27%	0
Odoribactersplanchnicus	14,64%	21,35%	18,00%	0
Ruminococcusbromii	15,02%	18,90%	16,96%	0
Bacteroidessp224	18,44%	13,22%	15,83%	0
Desulfovibriovulgaris	13,17%	17,45%	15,31%	0
_Eubacterium_cylindroides	10,99%	19,45%	15,22%	0
Bacteroidessp9142FAA	16,67%	12,82%	14,74%	0
Bacteroidesdorei	16,39%	12,48%	14,43%	0
Bacteroidesvulgatus	17,74%	10,76%	14,25%	0
Bacteroidessp3140A	13,20%	14,74%	13,97%	0
CoprococcusspART55_1	8,82%	17,43%	13,12%	0
Clostridiumhathewayi	7,43%	13,96%	10,69%	0
Tannerellaforsythia	10,62%	9,30%	9,96%	0
Roseburiahominis	10,16%	8,86%	9,51%	0
Acidaminococcusintestini	10,27%	6,61%	8,44%	0
RuminococcusspSR1_5	4,42%	10,31%	7,37%	0
Bacteroidesfragilis	7,36%	6,96%	7,16%	0
Collinsellaaerofaciens	6,74%	6,80%	6,77%	0
Dialisterinvisus	3,25%	8,88%	6,06%	0
Desulfovibriodesulfuricans	4,15%	7,34%	5,75%	0
Faecalibacteriumprausnitzii	2,33%	6,60%	4,47%	0
_Clostridium_difficile	2,25%	5,56%	3,91%	0
Clostridiumsymbiosum	4,73%	3,01%	3,87%	0
Clostridiumasparagiforme	0,45%	4,44%	2,44%	0
Parabacteroidesjohnsonii	0,21%	3,61%	1,91%	0
Bifidobacteriumbifidum	1,23%	1,09%	1,16%	0
Bacteroidesxylanisolvens	-0,37%	0,23%	-0,07%	0
butyrate_producingbacteriumSM4_1	-3,68%	2,77%	-0,46%	0
Acidaminococcusfermentans	-3,62%	-1,25%	-2,44%	0
Clostridiumsaccharolyticum	-4,71%	-0,57%	-2,64%	0
Coprococcuscomes	-1,46%	-5,60%	-3,53%	0
Bacteroidesplebeius	-2,05%	-5,09%	-3,57%	0
Bacteroidescellulosilyticus	-8,87%	-0,09%	-4,48%	0
Bacteroideshelcogenes	-6,64%	-3,72%	-5,18%	0
Bacteroidessp1114	-3,78%	-7,60%	-5,69%	0
Bacteroidessalanitronis	-5,42%	-8,19%	-6,81%	0

Supplementary Table S1B (continued)

Paludibacterpropionicigenes	**-6,79%**	**-6,83%**	**-6,81%**	0
Sutterellawadsworthensis	**-8,03%**	**-6,20%**	**-7,12%**	0
Holdemaniafiliformis	**-8,81%**	**-7,65%**	**-8,23%**	0
Prevotellacopri	**-6,17%**	**-10,31%**	**-8,24%**	0
Prevotellamelaninogenica	**-6,19%**	**-10,42%**	**-8,31%**	0
Bacteroidessp116	**-6,75%**	**-11,14%**	**-8,94%**	0
Bacteroidesthetaiotaomicron	**-8,30%**	**-10,02%**	**-9,16%**	0
Prevotellaintermedia	**-7,42%**	**-11,46%**	**-9,44%**	0
Clostridialesbacterium1747FAA	**-11,68%**	**-8,12%**	**-9,90%**	0
Prevotellaruminicola	**-8,66%**	**-12,50%**	**-10,58%**	0
Prevotelladenticola	**-9,09%**	**-12,62%**	**-10,86%**	0
BacteroidesspD20	**-12,46%**	**-9,63%**	**-11,05%**	0
Clostridiumbolteae	**-13,44%**	**-10,15%**	**-11,79%**	0
Paraprevotellaxylaniphila	**-16,46%**	**-11,23%**	**-13,85%**	0
Prevotelladentalis	**-12,82%**	**-16,48%**	**-14,65%**	0
Bacteroidesfinegoldii	**-17,11%**	**-12,54%**	**-14,83%**	0
Bacteroidessp4136	**-18,36%**	**-16,71%**	**-17,54%**	0
_Ruminococcus_obeum	**-21,60%**	**-15,61%**	**-18,60%**	0
Porphyromonasgingivalis	**-19,73%**	**-18,06%**	**-18,89%**	0
Bacteroidesuniformis	**-20,70%**	**-17,85%**	**-19,27%**	0
Phascolarctobacteriumsuccinatutens	**-16,66%**	**-22,73%**	**-19,70%**	0
Bacteroidesfluxus	**-21,88%**	**-20,22%**	**-21,05%**	0
butyrate_producingbacteriumSS3_4	**-21,48%**	**-21,62%**	**-21,55%**	0
Bifidobacteriumlongum	**-24,52%**	**-21,37%**	**-22,95%**	0
Roseburiaintestinalis	**-23,74%**	**-24,97%**	**-24,36%**	0
butyrate_producingbacteriumSSC_2	**-23,57%**	**-25,36%**	**-24,46%**	0
Megamonashypermegale	**-23,14%**	**-26,96%**	**-25,05%**	0
Roseburiainulinivorans	**-25,43%**	**-28,78%**	**-27,11%**	0
Parabacteroidesdistasonis	**-31,26%**	**-28,86%**	**-30,06%**	0
Eubacteriumrectale	**-30,49%**	**-34,19%**	**-32,34%**	0
Bacteroidesstercoris	**-34,41%**	**-36,10%**	**-35,25%**	0
_Ruminococcus_torques	**-39,53%**	**-38,45%**	**-38,99%**	0
Parabacteroidesmerdae	**-39,77%**	**-38,40%**	**-39,09%**	0
Ruminococcussp5139BFAA	**-41,63%**	**-40,89%**	**-41,26%**	0
Megasphaeraelsdenii	**-43,23%**	**-39,81%**	**-41,52%**	0
Coprococcuscatus	**-45,79%**	**-43,95%**	**-44,87%**	0,5
Bacteroidescaccae	**-44,41%**	**-46,34%**	**-45,38%**	0,5

Supplementary Table S2A

Elastic net regularization for different penalties (α = 1 corresponds to LASSO regresson, while α = 0.5 is a mix of LASSO and Ridge regression) and different numbers of folds (3, 5, 8 and 15). The number of repetitions was always 1000. Percentages show the proportion of repetitions where the respective predictor variable (from the set of genera) was present in the selected optimal model. Tables are sorted according to the mean of the percentages for terms appearing in models with delta BMI as the response and models with RWL as the response.

1000 repetitions

Sorted (mean)

Predictor variable importance: α = 1 / number of folds = 3

Predictor variable (genera)	deltaBMI	RWL	mean
Akkermansia	9,50%	17,30%	13,40%
**Symbiobacterium**	3,70%	23,00%	13,35%
Alistipes	9,70%	14,60%	12,15%
**Pseudoflavonifractor**	4,90%	3,90%	4,40%
Dorea	0,90%	0,00%	0,45%
Acidaminococcus	0,20%	0,40%	0,30%
Subdoligranulum	0,00%	0,40%	0,20%
Veillonella	0,00%	0,20%	0,10%
Eggerthella	0,10%	0,00%	0,05%
Parasutterella	0,10%	0,00%	0,05%
Staphylococcus	0,10%	0,00%	0,05%
Dialister	0,00%	0,00%	0,00%

Predictor variable importance: α = 1 / number of folds = 5

Predictor variable (genera)	deltaBMI	RWL	mean
Akkermansia	10,40%	46,10%	28,25%
**Symbiobacterium**	3,40%	51,80%	27,60%
Alistipes	10,40%	26,40%	18,40%
**Pseudoflavonifractor**	6,90%	15,90%	11,40%
Dorea	0,90%	0,00%	0,45%
Acidaminococcus	0,10%	0,00%	0,05%
Dialister	0,10%	0,00%	0,05%
Eggerthella	0,10%	0,00%	0,05%
Parasutterella	0,10%	0,00%	0,05%
Staphylococcus	0,10%	0,00%	0,05%
Subdoligranulum	0,00%	0,00%	0,00%
Veillonella	0,00%	0,00%	0,00%

Predictor variable importance: α = 1 / number of folds = 8

Predictor variable (genera)	deltaBMI	RWL	mean
Akkermansia	8,00%	79,70%	43,85%
**Symbiobacterium**	4,70%	82,60%	43,65%
**Pseudoflavonifractor**	6,30%	48,80%	27,55%
Alistipes	7,90%	21,60%	14,75%
Dorea	0,30%	0,00%	0,15%
Acidaminococcus	0,00%	0,00%	0,00%
Dialister	0,00%	0,00%	0,00%
Eggerthella	0,00%	0,00%	0,00%
Parasutterella	0,00%	0,00%	0,00%
Staphylococcus	0,00%	0,00%	0,00%
Subdoligranulum	0,00%	0,00%	0,00%
Veillonella	0,00%	0,00%	0,00%

Predictor variable importance: α = 1 / number of folds = 15

Predictor variable (genera)	deltaBMI	RWL	mean
Akkermansia	0,00%	100,00%	50,00%
**Pseudoflavonifractor**	0,00%	100,00%	50,00%
**Symbiobacterium**	0,00%	100,00%	50,00%
Acidaminococcus	0,00%	0,00%	0,00%
Alistipes	0,00%	0,00%	0,00%
Dialister	0,00%	0,00%	0,00%
Dorea	0,00%	0,00%	0,00%
Eggerthella	0,00%	0,00%	0,00%
Parasutterella	0,00%	0,00%	0,00%
Staphylococcus	0,00%	0,00%	0,00%
Subdoligranulum	0,00%	0,00%	0,00%
Veillonella	0,00%	0,00%	0,00%

Supplementary Table S2A (continued)

1000 repetitions			
**Sorted (mean)**			
Predictor variable importance: α = 0.75 / number of folds = 3			
Predictor variable (genera)	deltaBMI	RWL	mean
Alistipes	8,50%	20,40%	14,45%
**Symbiobacterium**	4,90%	21,10%	13,00%
Akkermansia	7,40%	15,40%	11,40%
**Pseudoflavonifractor**	3,60%	5,50%	4,55%
Dorea	0,30%	0,00%	0,15%
Veillonella	0,00%	0,20%	0,10%
Acidaminococcus	0,10%	0,00%	0,05%
Parasutterella	0,10%	0,00%	0,05%
Dialister	0,00%	0,00%	0,00%
Eggerthella	0,00%	0,00%	0,00%
Staphylococcus	0,00%	0,00%	0,00%
Subdoligranulum	0,00%	0,00%	0,00%

Predictor variable importance: α = 0.75 / number of folds = 5			
Predictor variable (genera)	deltaBMI	RWL	mean
Alistipes	6,70%	49,10%	27,90%
**Symbiobacterium**	3,90%	49,60%	26,75%
Akkermansia	6,60%	42,50%	24,55%
**Pseudoflavonifractor**	2,80%	23,20%	13,00%
Dorea	0,30%	0,00%	0,15%
Veillonella	0,00%	0,10%	0,05%
Acidaminococcus	0,00%	0,00%	0,00%
Dialister	0,00%	0,00%	0,00%
Eggerthella	0,00%	0,00%	0,00%
Parasutterella	0,00%	0,00%	0,00%
Staphylococcus	0,00%	0,00%	0,00%
Subdoligranulum	0,00%	0,00%	0,00%

Predictor variable importance: α = 0.75 / number of folds = 8			
Predictor variable (genera)	deltaBMI	RWL	mean
Alistipes	9,50%	83,10%	46,30%
**Symbiobacterium**	8,00%	83,10%	45,55%
Akkermansia	9,30%	79,70%	44,50%
**Pseudoflavonifractor**	6,10%	62,20%	34,15%
Acidaminococcus	0,00%	0,00%	0,00%
Dialister	0,00%	0,00%	0,00%
Dorea	0,00%	0,00%	0,00%
Eggerthella	0,00%	0,00%	0,00%
Parasutterella	0,00%	0,00%	0,00%
Staphylococcus	0,00%	0,00%	0,00%
Subdoligranulum	0,00%	0,00%	0,00%
Veillonella	0,00%	0,00%	0,00%

Predictor variable importance: α = 0.75 / number of folds = 15			
Predictor variable (genera)	deltaBMI	RWL	mean
Akkermansia	0,00%	100,00%	50,00%
Alistipes	0,00%	100,00%	50,00%
**Pseudoflavonifractor**	0,00%	100,00%	50,00%
**Symbiobacterium**	0,00%	100,00%	50,00%
Acidaminococcus	0,00%	0,00%	0,00%
Dialister	0,00%	0,00%	0,00%
Dorea	0,00%	0,00%	0,00%
Eggerthella	0,00%	0,00%	0,00%
Parasutterella	0,00%	0,00%	0,00%
Staphylococcus	0,00%	0,00%	0,00%
Subdoligranulum	0,00%	0,00%	0,00%
Veillonella	0,00%	0,00%	0,00%

Supplementary Table S2A (continued)

1000 repetitions			
**Sorted (mean)**			
Predictor variable importance: α = 0.5 / number of folds = 3			
Predictor variable (genera)	deltaBMI	RWL	mean
Alistipes	8,30%	23,40%	15,85%
**Symbiobacterium**	4,40%	23,40%	13,90%
Akkermansia	7,50%	20,20%	13,85%
**Pseudoflavonifractor**	2,50%	6,80%	4,65%
Parasutterella	0,70%	0,00%	0,35%
Subdoligranulum	0,10%	0,60%	0,35%
Acidaminococcus	0,00%	0,10%	0,05%
Eggerthella	0,10%	0,00%	0,05%
Veillonella	0,00%	0,10%	0,05%
Dialister	0,00%	0,00%	0,00%
Dorea	0,00%	0,00%	0,00%
Staphylococcus	0,00%	0,00%	0,00%

Predictor variable importance: α = 0.5 / number of folds = 5			
Predictor variable (genera)	deltaBMI	RWL	mean
Alistipes	6,10%	47,50%	26,80%
**Symbiobacterium**	3,80%	47,50%	25,65%
Akkermansia	5,90%	41,80%	23,85%
**Pseudoflavonifractor**	2,60%	18,40%	10,50%
Parasutterella	0,40%	0,00%	0,20%
Eggerthella	0,30%	0,00%	0,15%
Acidaminococcus	0,00%	0,00%	0,00%
Dialister	0,00%	0,00%	0,00%
Dorea	0,00%	0,00%	0,00%
Staphylococcus	0,00%	0,00%	0,00%
Subdoligranulum	0,00%	0,00%	0,00%
Veillonella	0,00%	0,00%	0,00%

Predictor variable importance: α = 0.5 / number of folds = 8			
Predictor variable (genera)	deltaBMI	RWL	mean
Alistipes	9,30%	79,80%	44,55%
**Symbiobacterium**	6,70%	79,80%	43,25%
Akkermansia	8,50%	76,30%	42,40%
**Pseudoflavonifractor**	5,00%	50,00%	27,50%
Eggerthella	0,70%	0,00%	0,35%
Acidaminococcus	0,00%	0,00%	0,00%
Dialister	0,00%	0,00%	0,00%
Dorea	0,00%	0,00%	0,00%
Parasutterella	0,00%	0,00%	0,00%
Staphylococcus	0,00%	0,00%	0,00%
Subdoligranulum	0,00%	0,00%	0,00%
Veillonella	0,00%	0,00%	0,00%

Predictor variable importance: α = 0.5 / number of folds = 15			
Predictor variable (genera)	deltaBMI	RWL	mean
Akkermansia	0,00%	100,00%	50,00%
Alistipes	0,00%	100,00%	50,00%
**Pseudoflavonifractor**	0,00%	100,00%	50,00%
**Symbiobacterium**	0,00%	100,00%	50,00%
Acidaminococcus	0,00%	0,00%	0,00%
Dialister	0,00%	0,00%	0,00%
Dorea	0,00%	0,00%	0,00%
Eggerthella	0,00%	0,00%	0,00%
Parasutterella	0,00%	0,00%	0,00%
Staphylococcus	0,00%	0,00%	0,00%
Subdoligranulum	0,00%	0,00%	0,00%
Veillonella	0,00%	0,00%	0,00%

Supplementary Table S2B

Elastic net regularization for different penalties (α = 1 corresponds to LASSO regresson, while α = 0.5 is a mix of LASSO and Ridge regression) and different numbers of folds (3, 5, 8 and 15). The number of repetitions was always 1000. Percentages show the proportion of repetitions where the respective predictor variable (from the set of species) was present in the selected optimal model. Tables are sorted according to the mean of the percentages for terms appearing in models with delta BMI as the response and models with RWL as the response.

1000 repetitions

Sorted (mean)

Predictor variable importance: α = 1 / number of folds = 3

Predictor variable (species)	deltaBMI	RWL	mean
**Alistipesfinegoldii**	100,00%	19,50%	59,75%
**Akkermansiamuciniphila**	9,00%	16,10%	12,55%
**Ethanoligenensharbinense**	1,50%	15,10%	8,30%
Bacteroidesovatus	1,30%	1,60%	1,45%
Roseburiahominis	1,30%	1,20%	1,25%
Bacteroideseggerthii	0,90%	1,40%	1,15%
Subdoligranulumvariabile	1,80%	0,00%	0,90%
X_Clostridium_difficile	0,40%	0,00%	0,20%
Coprococcuscomes	0,10%	0,00%	0,05%
Bacteroidesintestinalis	0,00%	0,00%	0,00%
Bacteroidessp3133FAA	0,00%	0,00%	0,00%
Bacteroidessp9142FAA	0,00%	0,00%	0,00%
Dorealongicatena	0,00%	0,00%	0,00%
Eubacteriumeligens	0,00%	0,00%	0,00%
Gordonibacterpamelaeae	0,00%	0,00%	0,00%
Oscillibactervalericigenes	0,00%	0,00%	0,00%
Phascolarctobacteriumsuccinatutens	0,00%	0,00%	0,00%
Pseudoflavonifractorcapillosus	0,00%	0,00%	0,00%

Predictor variable importance: α = 1 / number of folds = 5

Predictor variable (species)	deltaBMI	RWL	mean
**Alistipesfinegoldii**	100,00%	22,00%	61,00%
**Akkermansiamuciniphila**	10,10%	19,10%	14,60%
**Ethanoligenensharbinense**	0,30%	17,10%	8,70%
Bacteroidesovatus	0,20%	1,30%	0,75%
Roseburiahominis	0,30%	0,80%	0,55%
Bacteroideseggerthii	0,00%	1,00%	0,50%
Subdoligranulumvariabile	0,40%	0,00%	0,20%
Bacteroidesintestinalis	0,00%	0,00%	0,00%
Bacteroidessp3133FAA	0,00%	0,00%	0,00%
Bacteroidessp9142FAA	0,00%	0,00%	0,00%
Coprococcuscomes	0,00%	0,00%	0,00%
Dorealongicatena	0,00%	0,00%	0,00%
Eubacteriumeligens	0,00%	0,00%	0,00%
Gordonibacterpamelaeae	0,00%	0,00%	0,00%
Oscillibactervalericigenes	0,00%	0,00%	0,00%
Phascolarctobacteriumsuccinatutens	0,00%	0,00%	0,00%
Pseudoflavonifractorcapillosus	0,00%	0,00%	0,00%
X_Clostridium_difficile	0,00%	0,00%	0,00%

Predictor variable importance: α = 1 / number of folds = 8

Predictor variable (species)	deltaBMI	RWL	mean
**Alistipesfinegoldii**	100,00%	24,70%	62,35%
**Akkermansiamuciniphila**	7,40%	21,50%	14,45%
**Ethanoligenensharbinense**	0,00%	21,10%	10,55%
Bacteroideseggerthii	0,10%	1,90%	1,00%
Bacteroidesovatus	0,10%	1,90%	1,00%
Roseburiahominis	0,10%	1,80%	0,95%
Subdoligranulumvariabile	0,20%	0,00%	0,10%
X_Clostridium_difficile	0,10%	0,00%	0,05%
Bacteroidesintestinalis	0,00%	0,00%	0,00%
Bacteroidessp3133FAA	0,00%	0,00%	0,00%
Bacteroidessp9142FAA	0,00%	0,00%	0,00%
Coprococcuscomes	0,00%	0,00%	0,00%
Dorealongicatena	0,00%	0,00%	0,00%
Eubacteriumeligens	0,00%	0,00%	0,00%
Gordonibacterpamelaeae	0,00%	0,00%	0,00%
Oscillibactervalericigenes	0,00%	0,00%	0,00%
Phascolarctobacteriumsuccinatutens	0,00%	0,00%	0,00%
Pseudoflavonifractorcapillosus	0,00%	0,00%	0,00%

Predictor variable importance: α = 1 / number of folds = 15

Predictor variable (species)	deltaBMI	RWL	mean

Supplementary Table S2B (contiued)			
**Alistipesfinegoldii**	100,00%	0,00%	50,00%
**Akkermansiamuciniphila**	0,00%	0,00%	0,00%
Bacteroideseggerthii	0,00%	0,00%	0,00%
Bacteroidesintestinalis	0,00%	0,00%	0,00%
Bacteroidesovatus	0,00%	0,00%	0,00%
Bacteroidessp3133FAA	0,00%	0,00%	0,00%
Bacteroidessp9142FAA	0,00%	0,00%	0,00%
Coprococcuscomes	0,00%	0,00%	0,00%
Dorealongicatena	0,00%	0,00%	0,00%
**Ethanoligenensharbinense**	0,00%	0,00%	0,00%
Eubacteriumeligens	0,00%	0,00%	0,00%
Gordonibacterpamelaeae	0,00%	0,00%	0,00%
Oscillibactervalericigenes	0,00%	0,00%	0,00%
Phascolarctobacteriumsuccinatutens	0,00%	0,00%	0,00%
Pseudoflavonifractorcapillosus	0,00%	0,00%	0,00%
Roseburiahominis	0,00%	0,00%	0,00%
Subdoligranulumvariabile	0,00%	0,00%	0,00%
X_Clostridium_difficile	0,00%	0,00%	0,00%

1000 repetitions

Sorted (mean)

Predictor variable importance: α = 0.75 / number of folds = 3

Predictor variable (species)	deltaBMI	RWL	mean
**Alistipesfinegoldii**	15,50%	16,90%	16,20%
**Akkermansiamuciniphila**	6,70%	15,20%	10,95%
**Ethanoligenensharbinense**	0,70%	13,60%	7,15%
Bacteroidesovatus	0,20%	1,20%	0,70%
Roseburiahominis	0,20%	0,90%	0,55%
Bacteroideseggerthii	0,00%	0,90%	0,45%
Pseudoflavonifractorcapillosus	0,00%	0,50%	0,25%
Subdoligranulumvariabile	0,30%	0,00%	0,15%
Bacteroidesintestinalis	0,00%	0,00%	0,00%
Bacteroidessp3133FAA	0,00%	0,00%	0,00%
Bacteroidessp9142FAA	0,00%	0,00%	0,00%
Coprococcuscomes	0,00%	0,00%	0,00%
Dorealongicatena	0,00%	0,00%	0,00%
Eubacteriumeligens	0,00%	0,00%	0,00%
Gordonibacterpamelaeae	0,00%	0,00%	0,00%
Oscillibactervalericigenes	0,00%	0,00%	0,00%
Phascolarctobacteriumsuccinatutens	0,00%	0,00%	0,00%
X_Clostridium_difficile	0,00%	0,00%	0,00%

Predictor variable importance: α = 0.75 / number of folds = 5

Predictor variable (species)	deltaBMI	RWL	mean
**Alistipesfinegoldii**	16,20%	22,20%	19,20%
**Akkermansiamuciniphila**	6,10%	18,90%	12,50%
**Ethanoligenensharbinense**	0,10%	16,00%	8,05%
Bacteroidesovatus	0,00%	0,50%	0,25%
Bacteroideseggerthii	0,00%	0,30%	0,15%
Roseburiahominis	0,00%	0,30%	0,15%
Pseudoflavonifractorcapillosus	0,00%	0,10%	0,05%
Bacteroidesintestinalis	0,00%	0,00%	0,00%
Bacteroidessp3133FAA	0,00%	0,00%	0,00%
Bacteroidessp9142FAA	0,00%	0,00%	0,00%
Coprococcuscomes	0,00%	0,00%	0,00%
Dorealongicatena	0,00%	0,00%	0,00%
Eubacteriumeligens	0,00%	0,00%	0,00%
Gordonibacterpamelaeae	0,00%	0,00%	0,00%
Oscillibactervalericigenes	0,00%	0,00%	0,00%
Phascolarctobacteriumsuccinatutens	0,00%	0,00%	0,00%
Subdoligranulumvariabile	0,00%	0,00%	0,00%
X_Clostridium_difficile	0,00%	0,00%	0,00%

Predictor variable importance: α = 0.75 / number of folds = 8

Predictor variable (species)	deltaBMI	RWL	mean
**Alistipesfinegoldii**	16,30%	22,20%	19,25%
**Akkermansiamuciniphila**	6,60%	21,30%	13,95%
**Ethanoligenensharbinense**	0,00%	19,70%	9,85%
Bacteroideseggerthii	0,00%	0,60%	0,30%
Bacteroidesovatus	0,00%	0,60%	0,30%
Roseburiahominis	0,00%	0,50%	0,25%
Bacteroidesintestinalis	0,00%	0,00%	0,00%
Bacteroidessp3133FAA	0,00%	0,00%	0,00%
Bacteroidessp9142FAA	0,00%	0,00%	0,00%
Coprococcuscomes	0,00%	0,00%	0,00%
Dorealongicatena	0,00%	0,00%	0,00%
Eubacteriumeligens	0,00%	0,00%	0,00%

Supplementary Table S2B (contiued)

Gordonibacterpamelaeae	0,00%	0,00%	0,00%
Oscillibactervalericigenes	0,00%	0,00%	0,00%
Phascolarctobacteriumsuccinatutens	0,00%	0,00%	0,00%
Pseudoflavonifractorcapillosus	0,00%	0,00%	0,00%
Subdoligranulumvariabile	0,00%	0,00%	0,00%
X_Clostridium_difficile	0,00%	0,00%	0,00%

Predictor variable importance: α = 0.75 / number of folds = 15

Predictor variable (species)	deltaBMI	RWL	mean
**Akkermansiamuciniphila**	0,00%	0,00%	0,00%
**Alistipesfinegoldii**	0,00%	0,00%	0,00%
Bacteroideseggerthii	0,00%	0,00%	0,00%
Bacteroidesintestinalis	0,00%	0,00%	0,00%
Bacteroidesovatus	0,00%	0,00%	0,00%
Bacteroidessp3133FAA	0,00%	0,00%	0,00%
Bacteroidessp9142FAA	0,00%	0,00%	0,00%
Coprococcuscomes	0,00%	0,00%	0,00%
Dorealongicatena	0,00%	0,00%	0,00%
**Ethanoligenensharbinense**	0,00%	0,00%	0,00%
Eubacteriumeligens	0,00%	0,00%	0,00%
Gordonibacterpamelaeae	0,00%	0,00%	0,00%
Oscillibactervalericigenes	0,00%	0,00%	0,00%
Phascolarctobacteriumsuccinatutens	0,00%	0,00%	0,00%
Pseudoflavonifractorcapillosus	0,00%	0,00%	0,00%
Roseburiahominis	0,00%	0,00%	0,00%
Subdoligranulumvariabile	0,00%	0,00%	0,00%
X_Clostridium_difficile	0,00%	0,00%	0,00%

1000 repetitions

Sorted (mean)

Predictor variable importance: α = 0.5 / number of folds = 3

Predictor variable (species)	deltaBMI	RWL	mean
**Alistipesfinegoldii**	14,50%	100,00%	57,25%
**Akkermansiamuciniphila**	8,20%	14,80%	11,50%
**Ethanoligenensharbinense**	1,80%	14,80%	8,30%
Pseudoflavonifractorcapillosus	1,60%	7,80%	4,70%
Oscillibactervalericigenes	0,00%	2,50%	1,25%
Bacteroidesovatus	0,30%	1,90%	1,10%
Bacteroidesintestinalis	1,50%	0,00%	0,75%
Roseburiahominis	0,20%	0,80%	0,50%
Gordonibacterpamelaeae	0,90%	0,00%	0,45%
Subdoligranulumvariabile	0,90%	0,00%	0,45%
Bacteroideseggerthii	0,10%	0,70%	0,40%
Eubacteriumeligens	0,10%	0,20%	0,15%
Bacteroidessp3133FAA	0,10%	0,00%	0,05%
Bacteroidessp9142FAA	0,10%	0,00%	0,05%
Dorealongicatena	0,10%	0,00%	0,05%
Phascolarctobacteriumsuccinatutens	0,10%	0,00%	0,05%
Coprococcuscomes	0,00%	0,00%	0,00%
X_Clostridium_difficile	0,00%	0,00%	0,00%

Predictor variable importance: α = 0.5 / number of folds = 5

Predictor variable (species)	deltaBMI	RWL	mean
**Alistipesfinegoldii**	12,90%	100,00%	56,45%
**Akkermansiamuciniphila**	6,40%	14,80%	10,60%
**Ethanoligenensharbinense**	0,90%	14,80%	7,85%
Pseudoflavonifractorcapillosus	0,50%	6,60%	3,55%
Bacteroidesovatus	0,20%	0,50%	0,35%
Gordonibacterpamelaeae	0,70%	0,00%	0,35%
Bacteroidesintestinalis	0,40%	0,00%	0,20%
Oscillibactervalericigenes	0,00%	0,40%	0,20%
Roseburiahominis	0,10%	0,10%	0,10%
Subdoligranulumvariabile	0,20%	0,00%	0,10%
Bacteroideseggerthii	0,00%	0,10%	0,05%
Bacteroidessp3133FAA	0,00%	0,00%	0,00%
Bacteroidessp9142FAA	0,00%	0,00%	0,00%
Coprococcuscomes	0,00%	0,00%	0,00%
Dorealongicatena	0,00%	0,00%	0,00%
Eubacteriumeligens	0,00%	0,00%	0,00%
Phascolarctobacteriumsuccinatutens	0,00%	0,00%	0,00%
X_Clostridium_difficile	0,00%	0,00%	0,00%

Predictor variable importance: α = 0.5 / number of folds = 8

Predictor variable (species)	deltaBMI	RWL	mean
**Alistipesfinegoldii**	9,40%	100,00%	54,70%
**Akkermansiamuciniphila**	4,20%	14,50%	9,35%

Supplementary Table S2B (contiued)

**Ethanoligenensharbinense**	0,00%	14,50%	7,25%
Pseudoflavonifractorcapillosus	0,00%	4,20%	2,10%
Bacteroideseggerthii	0,00%	0,30%	0,15%
Bacteroidesovatus	0,00%	0,30%	0,15%
Roseburiahominis	0,00%	0,30%	0,15%
Bacteroidesintestinalis	0,00%	0,00%	0,00%
Bacteroidessp3133FAA	0,00%	0,00%	0,00%
Bacteroidessp9142FAA	0,00%	0,00%	0,00%
Coprococcuscomes	0,00%	0,00%	0,00%
Dorealongicatena	0,00%	0,00%	0,00%
Eubacteriumeligens	0,00%	0,00%	0,00%
Gordonibacterpamelaeae	0,00%	0,00%	0,00%
Oscillibactervalericigenes	0,00%	0,00%	0,00%
Phascolarctobacteriumsuccinatutens	0,00%	0,00%	0,00%
Subdoligranulumvariabile	0,00%	0,00%	0,00%
X_Clostridium_difficile	0,00%	0,00%	0,00%

Predictor variable importance: α = 0.5 / number of folds = 15

Predictor variable (species)	deltaBMI	RWL	mean
**Alistipesfinegoldii**	0,00%	100,00%	50,00%
**Akkermansiamuciniphila**	0,00%	0,00%	0,00%
Bacteroideseggerthii	0,00%	0,00%	0,00%
Bacteroidesintestinalis	0,00%	0,00%	0,00%
Bacteroidesovatus	0,00%	0,00%	0,00%
Bacteroidessp3133FAA	0,00%	0,00%	0,00%
Bacteroidessp9142FAA	0,00%	0,00%	0,00%
Coprococcuscomes	0,00%	0,00%	0,00%
Dorealongicatena	0,00%	0,00%	0,00%
**Ethanoligenensharbinense**	0,00%	0,00%	0,00%
Eubacteriumeligens	0,00%	0,00%	0,00%
Gordonibacterpamelaeae	0,00%	0,00%	0,00%
Oscillibactervalericigenes	0,00%	0,00%	0,00%
Phascolarctobacteriumsuccinatutens	0,00%	0,00%	0,00%
Pseudoflavonifractorcapillosus	0,00%	0,00%	0,00%
Roseburiahominis	0,00%	0,00%	0,00%
Subdoligranulumvariabile	0,00%	0,00%	0,00%
X_Clostridium_difficile	0,00%	0,00%	0,00%

Supplementary Table S3A

Calculation of importance indices (column D) and ranks (column E) and a combined index value (column F) to determine a list of the most important predictor variables from the set of genera in terms of the Monte Carlo method and a specific set of weights ("weights 2", see cells D9:D11).

	weight
model-weighted overall	10,00%
model-weighted df all	45,00%
av. variable importance	45,00%

idx	ct	final list (weighted; ordered) species	final index	final rank	final indValue	ch. rel. (%)
43	1	Megasphaera	1,0000	1,8250	0,9973	-8,13%
70	2	Symbiobacterium	0,8816	2,4750	0,9162	-27,19%
58	3	Marvinbryantia	0,5310	7,1250	0,6671	-5,34%
12	4	Blautia	0,4706	5,7250	0,6315	-2,85%
2	5	Alistipes	0,4627	9,5750	0,6135	-16,27%
80	6	Haemophilus	0,3517	17,4000	0,5137	-3,33%
9	7	Akkermansia	0,3458	21,4000	0,4966	-0,40%
22	8	Pseudoflavonifractor	0,3137	15,5250	0,4946	-4,84%
17	9	Oscillibacter	0,2908	18,1500	0,4706	-1,61%
40	10	Ethanoligenens	0,3386	30,1000	0,4631	-0,50%
41	11	Gordonibacter	0,2858	20,1250	0,4607	-2,41%
83	12	Human_endogenous_retroviruses	0,2765	21,6250	0,4496	-6,77%
46	13	Burkholderia	0,2122	17,8500	0,4192	-3,27%
50	14	Treponema	0,2104	21,6500	0,4055	-1,29%
78	15	Heliobacterium	0,2188	24,9250	0,4003	-2,06%
92	16	Corynebacterium	0,2082	25,2750	0,3920	-2,22%
64	17	Streptomyces	0,2048	27,2250	0,3833	-1,63%
79	18	Sphaerochaeta	0,1925	26,6250	0,3771	-2,50%
95	19	Propionibacterium	0,2219	35,4250	0,3677	-1,00%
36	20	Eggerthella	0,2230	36,7750	0,3640	-1,12%
89	21	Bordetella	0,1789	29,1000	0,3599	-0,23%
31	22	unclassified_Erysipelotrichaceae	0,2092	35,4750	0,3591	-0,27%
42	23	Dorea	0,1808	30,0250	0,3581	-2,46%
85	24	Deinococcus	0,1832	33,1750	0,3493	-0,22%
35	25	Streptococcus	0,1669	30,1250	0,3485	-0,28%
54	26	Slackia	0,1884	34,7750	0,3475	-0,87%
90	27	Acidovorax	0,1595	29,8500	0,3445	-0,31%
82	28	Dysgonomonas	0,1599	30,2500	0,3434	-2,82%
91	29	Aeromonas	0,1557	32,3500	0,3337	-0,57%
62	30	Desulfitobacterium	0,1603	33,8500	0,3318	-0,88%
99	31	Mycobacterium	0,1856	39,8500	0,3289	-1,32%
39	32	Enterococcus	0,1779	39,6000	0,3246	-2,22%
18	33	Megamonas	0,1594	38,0500	0,3173	-0,46%
75	34	Ralstonia	0,1480	36,2000	0,3159	-0,09%
37	35	Porphyromonas	0,1551	37,7250	0,3156	-0,02%
11	36	Bifidobacterium	0,1712	41,0000	0,3155	-0,46%
30	37	unclassified_Ruminococcaceae	0,1735	41,9000	0,3140	-0,26%
94	38	Anaeromyxobacter	0,1524	37,8750	0,3132	-0,88%
66	39	Fretibacterium	0,1657	41,4000	0,3105	-0,30%
65	40	Parasutterella	0,1788	44,3250	0,3096	-0,88%
102	41	Spirochaeta	0,1606	41,4750	0,3068	-0,97%
48	42	Holdemania	0,1617	42,6000	0,3039	-2,28%
57	43	Desulfotomaculum	0,1512	42,5750	0,2969	-1,45%
10	44	Clostridium	0,1630	46,2750	0,2926	-0,63%
87	45	Cupriavidus	0,1382	41,8250	0,2908	-1,07%
51	46	Pseudomonas	0,1349	42,1000	0,2877	-0,70%
71	47	Enterobacter	0,1381	43,3500	0,2857	-1,42%
1	48	Bacteroides	0,1449	45,9500	0,2816	-0,18%
60	49	Geobacter	0,1404	45,2000	0,2811	-2,95%
26	50	Collinsella	0,1306	45,7250	0,2728	-1,73%
23	51	Paraprevotella	0,1341	47,8500	0,2681	-1,08%
81	52	unclassified_Burkholderiales_miscellaneous	0,1578	53,5250	0,2652	-5,04%
5	53	Parabacteroides	0,1416	54,3000	0,2519	-0,68%
101	54	Geobacillus	0,1205	50,5500	0,2501	-1,88%
53	55	Olsenella	0,1382	55,5500	0,2455	-1,08%
19	56	Subdoligranulum	0,1228	53,2500	0,2428	-0,08%
52	57	Paenibacillus	0,1245	53,6500	0,2426	-3,27%
38	58	Anaerotruncus	0,1072	52,5500	0,2347	-0,61%
13	59	unclassified_Clostridiales_miscellaneous	0,1285	57,3000	0,2332	-0,89%
33	60	unclassified_Peptostreptococcaceae	0,1255	57,3250	0,2312	-3,37%
98	61	Rhizobium	0,1100	56,5500	0,2234	-4,74%
61	62	Coriobacterium	0,1117	60,1000	0,2128	-2,35%

Supplementary Table S3A (continued)

6	63	Prevotella	0,1129	61,8500	0,2078	-0,71%
47	64	unclassified_sequences	0,1040	60,5000	0,2063	-1,96%
4	65	Eubacterium	0,1176	64,4750	0,2023	-2,15%
14	66	Odoribacter	0,1026	62,7750	0,1979	-0,61%
100	67	Bradyrhizobium	0,0996	62,5250	0,1967	-0,96%
74	68	environmental_samples_Bacteria	0,1032	63,8250	0,1948	-1,38%
77	69	Azospirillum	0,0978	63,5500	0,1921	-0,23%
21	70	unclassified_Lachnospiraceae	0,1263	69,4500	0,1917	-0,77%
44	71	Bacillus	0,1115	66,9000	0,1902	-0,27%
97	72	Klebsiella	0,0982	64,3750	0,1897	-0,13%
32	73	Butyrivibrio	0,1010	65,0000	0,1895	-0,51%
45	74	Selenomonas	0,0911	63,3000	0,1885	-0,31%
59	75	Barnesiella	0,1135	68,0000	0,1879	-0,21%
29	76	Tannerella	0,0862	62,6000	0,1875	-1,44%
34	77	Escherichia	0,1039	67,0000	0,1848	-0,56%
96	78	Syntrophobotulus	0,0938	65,2750	0,1838	-1,63%
49	79	Paludibacter	0,0997	67,3750	0,1808	-3,43%
88	80	Oribacterium	0,0891	67,1250	0,1746	-1,14%
93	81	Rhodospirillum	0,0853	66,9500	0,1726	-10,36%
72	82	Flavobacterium	0,0822	71,7500	0,1547	-2,26%
67	83	Bacteroides_pectinophilus	0,0949	75,3750	0,1512	-1,52%
15	84	Coprococcus	0,0834	73,7500	0,1489	-6,42%
56	85	Lactobacillus	0,0800	75,9500	0,1394	-6,24%
7	86	Roseburia	0,0856	79,7250	0,1307	-4,46%
68	87	Capnocytophaga	0,0797	80,3000	0,1248	-5,79%
73	88	Cellulosilyticum	0,0792	82,3750	0,1176	-0,21%
27	89	Bilophila	0,0764	81,9000	0,1174	-8,59%
24	90	Phascolarctobacterium	0,0769	85,0500	0,1073	-11,96%
69	91	Pedobacter	0,0689	87,3250	0,0944	-1,41%
8	92	Ruminococcus	0,0704	88,0250	0,0931	-0,16%
20	93	Dialister	0,0642	86,8250	0,0930	-6,25%
86	94	Sphingobacterium	0,0717	90,1000	0,0872	-6,67%
76	95	Staphylococcus	0,0708	91,6750	0,0813	-2,83%
3	96	Faecalibacterium	0,0702	92,2500	0,0790	-3,77%
55	97	Campylobacter	0,0620	91,5000	0,0761	-15,68%
16	98	Acidaminococcus	0,0600	94,7000	0,0641	-17,53%
84	99	Chitinophaga	0,0589	97,9000	0,0529	-8,86%
28	100	Sutterella	0,0593	99,4000	0,0482	-5,52%
25	101	Desulfovibrio	0,0582	99,9750	0,0455	-9,06%
63	102	Veillonella	0,0572	101,0250	0,0414	

Supplementary Table S3B

Calculation of importance indices (column D) and ranks (column E) and a combined index value (column F) to determine a list of the most important predictor variables from the set of species in terms of the Monte Carlo method and a specific set of weights ("weights 2", see cells D9:D11).

	weight
model-weighted overall	10,00%
model-weighted df all	45,00%
av. variable importance	45,00%

idx	ct	final list (weighted; ordered) species	final index	final rank	final indValue	ch. rel. (%)
8	1	Alistipesfinegoldii	0,9329	1,9500	0,9522	-23,25%
59	2	Bacteroidescaccae	0,6193	5,8000	0,7308	-5,98%
20	3	AlistipesspHGB5	0,5531	5,6750	0,6871	-10,24%
12	4	Roseburiaintestinalis	0,4920	14,8750	0,6167	-2,07%
11	5	Akkermansiamuciniphila	0,4507	10,2750	0,6040	-6,57%
37	6	Coprococcuscatus	0,3983	11,7500	0,5643	-1,10%
34	7	Megamonashypermegale	0,3933	12,6500	0,5581	-9,55%
67	8	Bacteroidesintestinalis	0,3542	21,1250	0,5048	-0,31%
43	9	Pseudoflavonifractorcapillosus	0,3357	17,7750	0,5032	-0,29%
89	10	Ethanoligenensharbinense	0,3384	18,8000	0,5017	-0,61%
33	11	Oscillibactervalericigenes	0,3506	22,2750	0,4987	-3,00%
90	12	Gordonibacterpamelaeae	0,3129	19,1250	0,4837	-4,11%
98	13	Megasphaeraelsdenii	0,2928	21,1250	0,4638	-1,81%
40	14	Bacteroidesstercoris	0,2656	18,1000	0,4554	-2,65%
26	15	X_Ruminococcus_obeum	0,2808	25,0000	0,4434	-1,01%
35	16	Subdoligranulumvariabile	0,2686	23,8750	0,4389	-0,88%
52	17	Collinsellaaerofaciens	0,2636	24,0250	0,4350	-0,69%
31	18	Bifidobacteriumlongum	0,2844	29,2750	0,4321	-1,72%
88	19	Ruminococcussp5139BFAA	0,2567	25,8500	0,4246	-5,05%
22	20	X_Ruminococcus_torques	0,2303	27,0500	0,4032	-0,48%
79	21	Bifidobacteriumbifidum	0,2416	30,0000	0,4012	-0,54%
76	22	Bacteroidescoprophilus	0,2102	24,1500	0,3991	-1,99%
75	23	Bacteroidescoprocola	0,2156	27,7500	0,3911	-2,14%
44	24	Bacteroidesovatus	0,2326	33,8750	0,3828	-0,89%
16	25	Alistipesputredinis	0,2116	30,5750	0,3793	-2,15%
71	26	X_Clostridium_difficile	0,2021	31,1500	0,3712	-0,86%
54	27	RuminococcaceaebacteriumD16	0,2036	32,4500	0,3680	-2,41%
15	28	Eubacteriumeligens	0,1709	28,4250	0,3591	-0,39%
82	29	Eggerthellalenta	0,1923	33,3000	0,3577	-0,01%
45	30	Prevotelladentalis	0,1678	28,2250	0,3577	-1,74%
66	31	Clostridiumbolteae	0,2277	42,6000	0,3515	-0,20%
69	32	Ruminococcuschampanellensis	0,1817	33,2750	0,3508	-5,93%
64	33	Parabacteroidesmerdae	0,1634	35,9500	0,3300	-2,18%
21	34	butyrate_producingbacteriumSS3_4	0,1901	43,7250	0,3228	-3,05%
73	35	Clostridiumleptum	0,1466	37,7500	0,3129	-1,91%
13	36	Prevotellacopri	0,1251	35,1500	0,3070	-1,38%
58	37	Roseburiainulinivorans	0,1291	37,3000	0,3027	-0,42%
36	38	Prevotellaruminicola	0,1457	41,1500	0,3015	-0,74%
51	39	Tannerellaforsythia	0,1500	42,7250	0,2992	-2,70%
14	40	Odoribactersplanchnicus	0,1234	39,7250	0,2911	-2,52%
32	41	Bacteroidesuniformis	0,1144	40,1500	0,2838	-1,02%
4	42	Eubacteriumrectale	0,1406	46,4750	0,2809	-0,02%
100	43	Clostridiumhathewayi	0,1793	54,5250	0,2808	-3,62%
61	44	Bacteroidessp4136	0,1246	46,3500	0,2707	-1,39%
102	45	Holdemaniafiliformis	0,1117	44,8500	0,2669	-0,79%
101	46	Eubacteriumlimosum	0,1313	49,5750	0,2648	-1,14%
104	47	Paludibacterpropionicigenes	0,1254	49,2750	0,2618	-0,30%
93	48	Clostridiumsymbiosum	0,1502	54,6750	0,2610	-0,30%
103	49	unculturedorganism	0,1164	47,9000	0,2602	-0,19%
86	50	Bacteroidessp3133FAA	0,1130	47,3500	0,2597	-4,71%
49	51	Prevotellamelaninogenica	0,1051	49,5250	0,2475	-0,15%
6	52	Parabacteroidesdistasonis	0,1091	50,4750	0,2471	-0,69%
53	53	Prevotelladenticola	0,1044	50,0250	0,2454	-1,28%
62	54	butyrate_producingbacteriumSM4_1	0,1716	64,9500	0,2423	-1,15%
74	55	Prevotellaintermedia	0,1050	52,0000	0,2395	-0,07%
56	56	butyrate_producingbacteriumSSC_2	0,1024	51,5000	0,2393	-2,52%
92	57	Butyrivibrioproteoclasticus	0,1084	54,6250	0,2333	-0,07%
28	58	Bacteroideseggerthii	0,1119	55,4000	0,2331	-3,24%
80	59	ClostridiumspSY8519	0,1062	56,5750	0,2255	-2,92%
65	60	Lachnospiraceaebacterium3157FAACT1	0,1009	57,5250	0,2189	-1,87%
84	61	Anaerotruncuscolihominis	0,0996	58,5250	0,2149	-2,03%
63	62	X_Eubacterium_cylindroides	0,1023	60,4500	0,2105	-0,64%

Supplementary Table 3B (continued)

23	63	Roseburiahominis	0,0939	59,1250	0,2092	-0,24%
57	64	Ruminococcusalbus	0,0997	60,4750	0,2087	-3,58%
19	65	Eubacteriumsiraeum	0,0951	61,8500	0,2012	-1,50%
17	66	Bacteroidessalanitronis	0,1555	75,3250	0,1982	-0,91%
2	67	Faecalibacteriumprausnitzii	0,0850	61,2500	0,1964	-0,77%
78	68	Coprococcuscomes	0,1183	68,6250	0,1949	-2,40%
1	69	Bacteroidesvulgatus	0,0814	62,4250	0,1902	-0,12%
106	70	Dorealongicatena	0,0947	65,2500	0,1900	-0,14%
91	71	Bacteroidessp3140A	0,1003	66,5000	0,1897	-3,54%
94	72	Porphyromonasgingivalis	0,0835	65,1000	0,1830	-5,40%
68	73	Escherichiacoli	0,0836	68,2000	0,1731	-0,28%
46	74	Paraprevotellaxylaniphila	0,0754	66,6500	0,1726	-7,80%
9	75	Alistipesshahii	0,0787	71,5250	0,1592	-1,61%
96	76	Bacteroidesclarus	0,0744	71,4250	0,1566	-3,29%
7	77	Bacteroidesfragilis	0,0691	71,9250	0,1514	-0,21%
18	78	Ruminococcusbromii	0,0741	73,0750	0,1511	-2,59%
95	79	Clostridialesbacterium1747FAA	0,0767	74,8250	0,1472	-7,68%
25	80	BacteroidesspD20	0,0661	76,1500	0,1359	-1,60%
85	81	Bacteroidesfluxus	0,0747	78,6000	0,1337	-1,55%
87	82	Bacteroidessp224	0,0738	79,0750	0,1316	-0,50%
5	83	Bacteroidesthetaiotaomicron	0,0662	77,7000	0,1310	-1,14%
42	84	Dialisterinvisus	0,0631	77,5250	0,1295	-0,76%
48	85	Bacteroidesdorei	0,0677	78,7750	0,1285	-1,04%
30	86	Clostridiumsaccharolyticum	0,0673	79,1250	0,1272	-5,13%
70	87	Bacteroidessp9142FAA	0,0676	81,2000	0,1206	-7,85%
41	88	Bacteroidescellulosilyticus	0,0682	84,2750	0,1112	-3,77%
47	89	Phascolarctobacteriumsuccinatutens	0,0665	85,2250	0,1070	-0,46%
99	90	Desulfovibriovulgaris	0,0648	85,0250	0,1065	-2,18%
55	91	Bilophilawadsworthia	0,0655	85,9000	0,1042	-0,32%
77	92	Clostridiumasparagiforme	0,0669	86,3000	0,1038	-1,31%
83	93	Acidaminococcusfermentans	0,0617	85,6500	0,1025	-0,08%
29	94	Bifidobacteriumadolescentis	0,0660	86,5500	0,1024	-1,39%
39	95	CoprococcusspART55_1	0,0653	86,8500	0,1010	-0,13%
72	96	Bacteroidesfinegoldii	0,0622	86,2500	0,1008	-0,49%
97	97	Parabacteroidesjohnsonii	0,0641	86,8000	0,1003	-13,41%
10	98	Bacteroideshelcogenes	0,0600	90,1250	0,0869	-0,36%
3	99	Bacteroidesxylanisolvens	0,0641	91,0750	0,0866	-0,22%
24	100	RuminococcusspSR1_5	0,0644	91,2000	0,0864	-1,12%
50	101	Sutterellawadsworthensis	0,0634	91,3000	0,0854	-13,47%
81	102	Desulfovibriodesulfuricans	0,0600	94,1750	0,0739	-5,73%
27	103	Bacteroidesplebeius	0,0568	94,8250	0,0697	-4,55%
38	104	Acidaminococcusintestini	0,0577	96,0000	0,0665	-20,01%
105	105	Bacteroidessp116	0,0557	99,7250	0,0532	-27,61%
60	106	Bacteroidessp1114	0,0529	103,7250	0,0385	

Supplementary Table S4A

Number of terms, degrees of freedom (df), AICc, adjusted R^2 and relative standard error of the optimal linear regression models with one to five terms containing proportions of genera as predictor variables for both delta BMI (left) and RWL (right).

Mean squared errors and R^2-values were estimated for an independent test data set by performing a cross-validation on the original data set with 3, 5, 8 and 15 folds, and calculating mean squared errors and R^2-values from the averaged predictions.

	**delta BMI**				
**Predictor variables**	Symbio-bacterium	Mega-sphaera	Marvin-bryantia	Blautia	Blautia
		Symbio-bacterium	Mega-sphaera	Marvin-bryantia	Marvin-bryantia
			Symbio-bacterium	Mega-sphaera	Mega-sphaera
				Symbio-bacterium	Oscillibacter
					Symbio-bacterium
**number of terms**	1	2	3	4	5
df	3	4	5	6	7
**AICc**	90,1	81,1	77,2	74,9	77,8
**adjusted R^2**	0,1985	0,6297	0,7722	0,8539	0,8803
**relative standard error**	47,32%	24,89%	34,28%	37,40%	29,27%
		24,09%	21,45%	22,13%	21,24%
			16,65%	20,73%	17,79%
				12,65%	55,85%
					21,18%
**mean squared error**	**13,74**	**5,86**	**3,30**	**1,93**	**1,42**
**mean squared error from CV / 3 folds**	18,21	34,86	13,78	5,65	6,42
**mean squared error from CV / 5 folds**	18,09	34,77	14,54	5,89	6,61
**mean squared error from CV / 8 folds**	18,19	35,24	14,73	6,01	6,52
**mean squared error from CV / 15 folds**	18,11	34,96	14,79	6,09	6,63
**R^2**	**0,2557**	**0,6826**	**0,8210**	**0,8956**	**0,9230**
**R^2 from CV / 3 folds**	N/A	N/A	0,2535	0,6942	0,6525
**R^2 from CV / 5 folds**	N/A	N/A	0,2126	0,6808	0,6423
**R^2 from CV / 8 folds**	N/A	N/A	0,2022	0,6743	0,6469
**R^2 from CV / 15 folds**	N/A	N/A	0,1989	0,6700	0,6411

	**RWL**				
**Predictor variables**	Symbio-bacterium	Mega-sphaera	Marvin-bryantia	Blautia	Blautia
		Symbio-bacterium	Mega-sphaera	Marvin-bryantia	Marvin-bryantia
			Symbio-bacterium	Mega-sphaera	Mega-sphaera
				Symbio-bacterium	Oscillibacter
					Symbio-bacterium
**number of terms**	1	2	3	4	5
df	3	4	5	6	7
**AICc**	114,7	103,9	100,3	101,2	105,9
**adjusted R^2**	0,2956	0,7129	0,8194	0,8574	0,8682
**relative standard error**	38,14%	22,42%	35,19%	50,43%	41,25%
		19,10%	19,35%	26,67%	27,13%
			14,23%	23,85%	21,91%
				12,27%	74,13%
					23,71%
**mean squared error**	**71,21**	**26,79**	**15,45**	**11,09**	**9,22**
**mean squared error from CV / 3 folds**	92,31	155,65	67,51	33,75	30,88
**mean squared error from CV / 5 folds**	92,72	155,49	67,58	34,08	31,71
**mean squared error from CV / 8 folds**	92,31	157,09	67,85	34,17	31,87
**mean squared error from CV / 15 folds**	92,35	155,32	65,88	33,79	32,05
**R^2**	**0,3459**	**0,7539**	**0,8581**	**0,8981**	**0,9153**
**R^2 from CV / 3 folds**	N/A	N/A	0,3798	0,6900	0,7163
**R^2 from CV / 5 folds**	N/A	N/A	0,3792	0,6869	0,7087
**R^2 from CV / 8 folds**	N/A	N/A	0,3767	0,6861	0,7072
**R^2 from CV / 15 folds**	N/A	N/A	0,3949	0,6896	0,7056

Supplementary Table S4B

Number of terms, degrees of freedom (df), AICc, adjusted R^2 and relative standard error of the optimal linear regression models with one to five terms containing proportions of species as predictor variables for both delta BMI (left) and RWL (right).

Mean squared errors and R^2-values were estimated for an independent test data set by performing a cross-validation on the original data set with 3, 5, 8 and 15 folds, and calculating mean squared errors and R^2-values from the averaged predictions.

	delta BMI				
**Predictor variables**	Alistipes finegoldii	Alistipes finegoldii<br>Bacteroides caccae	Alistipes finegoldii<br>Bacteroides caccae<br>Roseburia intestinalis	Alistipes finegoldii<br>Bacteroides caccae<br>Bacteroides stercoris<br>Roseburia intestinalis	Alistipes finegoldii<br>Alistipes spHGB5<br>Bacteroides caccae<br>Bacteroides stercoris<br>Roseburia intestinalis
**number of terms**	1	2	3	4	5
df	3	4	5	6	7
**AICc**	87,3	78,7	71,9	66,8	72,6
**adjusted R^2**	0,3348	0,6843	0,8394	0,9148	0,9156
**relative standard error**	35,25%	20,59%<br>25,49%	14,57%<br>16,02%<br>28,18%	12,21%<br>11,34%<br>30,54%<br>19,12%	40,44%<br>95,36%<br>12,93%<br>29,85%<br>18,73%
**mean squared error**	**11,41**	**5,00**	**2,33**	**1,12**	**1,00**
**mean squared error from CV / 3 folds**	16,85	7,04	4,47	3,74	4,88
**mean squared error from CV / 5 folds**	17,17	6,79	3,95	3,70	4,13
**mean squared error from CV / 8 folds**	17,21	6,69	3,87	3,68	4,71
**mean squared error from CV / 15 folds**	17,24	6,67	3,90	3,74	5,58
**R^2**	**0,3823**	**0,7294**	**0,8738**	**0,9391**	**0,9457**
**R^2 from CV / 3 folds**	N/A	0,6188	0,7577	0,7975	0,7358
**R^2 from CV / 5 folds**	N/A	0,6326	0,7862	0,7997	0,7763
**R^2 from CV / 8 folds**	N/A	0,6377	0,7906	0,8005	0,7452
**R^2 from CV / 15 folds**	N/A	0,6388	0,7887	0,7975	0,6977

	RWL				
**Predictor variables**	Alistipes finegoldii	Alistipes finegoldii<br>Bacteroides caccae	Alistipes finegoldii<br>Bacteroides caccae<br>Roseburia intestinalis	Alistipes finegoldii<br>Bacteroides caccae<br>Bacteroides stercoris<br>Roseburia intestinalis	Alistipes finegoldii<br>Bacteroides caccae<br>Bacteroides stercoris<br>Ethanolig. harbinense<br>Roseburia intestinalis
**number of terms**	1	2	3	4	5
df	3	4	5	6	7
**AICc**	114,7	106,4	99,4	91	95,9
**adjusted R^2**	0,2955	0,6599	0,8304	0,9273	0,9323
**relative standard error**	38,15%	22,18%<br>25,88%	15,54%<br>16,05%<br>27,67%	11,90%<br>10,18%<br>25,26%<br>16,78%	12,25%<br>10,68%<br>32,15%<br>75,74%<br>18,43%
**mean squared error**	**71,22**	**31,74**	**14,51**	**5,65**	**4,73**
**mean squared error from CV / 3 folds**	105,70	43,40	24,41	16,70	15,74
**mean squared error from CV / 5 folds**	106,59	43,08	22,74	17,50	15,82
**mean squared error from CV / 8 folds**	107,37	42,56	22,45	17,98	16,61
**mean squared error from CV / 15 folds**	107,76	42,29	23,00	18,47	17,46
**R^2**	**0,3458**	**0,7085**	**0,8667**	**0,9481**	**0,9565**
**R^2 from CV / 3 folds**	N/A	0,6014	0,7758	0,8466	0,8554
**R^2 from CV / 5 folds**	N/A	0,6042	0,7911	0,8393	0,8547
**R^2 from CV / 8 folds**	N/A	0,6090	0,7938	0,8348	0,8474
**R^2 from CV / 15 folds**	N/A	0,6115	0,7887	0,8303	0,8396

Supplementary Table S5A

Predicted weight loss, calculated as delta BMI, including classification and relative as well as absolute deviations of the predicted weight loss to the observed weight loss. Values are calculated for each patient under the optimal linear regression models with one to five linear terms containing predictor variables from the final set of selected genera of Table 6. Predictions for patients, numbered from 1 – 15 were classified as "correct(+)" in case significant weight loss occurred and was predicted, as "correct(-)" in case significant weight loss did not occur and was predicted as not occurring, as "overpredicted" in case a model predicted significant weight loss but it did not occur, and as "underpredicted" in case a model predicted no significant weight loss but significant weight loss occurred . The threshold for a significant weight loss was chosen as 4.8 in case of delta BMI.

Absolute deviations are the differences between predicted and observed BMI.

Relative deviations are absolute deviations divided by observed BMI.

DS	ID	Experimental deltaBMI	Predicted deltaBMI 1 term model	Predicted deltaBMI 2 terms model	Predicted deltaBMI 3 terms model	Predicted deltaBMI 4 terms model	Predicted deltaBMI 5 terms model
1	AS44	3,493755	0,958803	1,748966	2,891745	3,231218	3,566491
2	AS45	-4,162065	3,934257	-6,030012	-4,841759	-4,143036	-4,032864
3	AS50	5,497006	2,105900	3,574203	4,355042	3,805976	3,970089
4	AS51	0,730460	2,302431	3,179765	0,881268	0,568658	0,076206
5	AS53	-4,514210	0,878587	0,177932	-2,753783	-3,481998	-2,610879
6	AS56	7,136672	4,225984	6,331825	8,082186	8,429783	8,940461
7	AS58	4,577500	2,751644	2,911705	0,974659	3,203305	3,291153
8	AS60	8,923260	5,084506	5,844778	6,491457	7,205909	7,684455
9	AS61	5,119567	7,784050	6,766043	5,481692	2,962458	3,545577
10	AS62	5,900277	5,655660	6,594179	5,510664	6,009453	5,730281
11	AS63	3,689796	3,963359	4,830592	5,980822	4,491976	5,102556
12	AS64	5,607585	2,282377	3,728585	4,431196	5,133634	5,079464
13	AS65	-1,212121	1,648666	1,161091	2,362797	0,721703	-1,263880
14	AS66	4,996348	7,738965	8,379718	6,948426	7,993532	6,905266
15	AS68	11,423324	5,891541	8,007625	10,410287	11,074314	11,223004

					deltaBMI	threshold:	4,8
DS	ID	Experimental deltaBMI	Classification deltaBMI 1 term model	Classification deltaBMI 2 terms model	Classification deltaBMI 3 terms model	Classification deltaBMI 4 terms model	Classification deltaBMI 5 terms model
1	AS44	3,493755	correct(-)	correct(-)	correct(-)	correct(-)	correct(-)
2	AS45	-4,162065	correct(-)	correct(-)	correct(-)	correct(-)	correct(-)
3	AS50	5,497006	underpredicted	underpredicted	underpredicted	underpredicted	underpredicted
4	AS51	0,730460	correct(-)	correct(-)	correct(-)	correct(-)	correct(-)
5	AS53	-4,514210	correct(-)	correct(-)	correct(-)	correct(-)	correct(-)
6	AS56	7,136672	underpredicted	correct(+)	correct(+)	correct(+)	correct(+)
7	AS58	4,577500	correct(-)	correct(-)	correct(-)	correct(-)	correct(-)
8	AS60	8,923260	correct(+)	correct(+)	correct(+)	correct(+)	correct(+)
9	AS61	5,119567	correct(+)	correct(+)	correct(+)	underpredicted	underpredicted
10	AS62	5,900277	correct(+)	correct(+)	correct(+)	correct(+)	correct(+)
11	AS63	3,689796	correct(-)	overpredicted	overpredicted	correct(-)	overpredicted
12	AS64	5,607585	underpredicted	underpredicted	underpredicted	correct(+)	correct(+)
13	AS65	-1,212121	correct(-)	correct(-)	correct(-)	correct(-)	correct(-)
14	AS66	4,996348	correct(+)	correct(+)	correct(+)	correct(+)	correct(+)
15	AS68	11,423324	correct(+)	correct(+)	correct(+)	correct(+)	correct(+)
		correct(+)	5	6	6	6	6
		correct(-)	7	6	6	7	6
		underpredicted	3	2	2	2	2
		overpredicted	0	1	1	0	1
		sum correct	12	12	12	13	12
		sum incorrect	3	3	3	2	3

DS	ID	Experimental deltaBMI	Abs. deviation deltaBMI 1 term model	Abs. deviation deltaBMI 2 terms model	Abs. deviation deltaBMI 3 terms model	Abs. deviation deltaBMI 4 terms model	Abs. deviation deltaBMI 5 terms model
1	AS44	3,493755	-2,5350	-1,7448	-0,6020	-0,2625	0,0727
2	AS45	-4,162065	8,0963	-1,8679	-0,6797	0,0190	0,1292
3	AS50	5,497006	-3,3911	-1,9228	-1,1420	-1,6910	-1,5269
4	AS51	0,730460	1,5720	2,4493	0,1508	-0,1618	-0,6543
5	AS53	-4,514210	5,3928	4,6921	1,7604	1,0322	1,9033
6	AS56	7,136672	-2,9107	-0,8048	0,9455	1,2931	1,8038
7	AS58	4,577500	-1,8259	-1,6658	-3,6028	-1,3742	-1,2863
8	AS60	8,923260	-3,8388	-3,0785	-2,4318	-1,7174	-1,2388
9	AS61	5,119567	2,6645	1,6465	0,3621	-2,1571	-1,5740
10	AS62	5,900277	-0,2446	0,6939	-0,3896	0,1092	-0,1700
11	AS63	3,689796	0,2736	1,1408	2,2910	0,8022	1,4128
12	AS64	5,607585	-3,3252	-1,8790	-1,1764	-0,4740	-0,5281
13	AS65	-1,212121	2,8608	2,3732	3,5749	1,9338	-0,0518
14	AS66	4,996348	2,7426	3,3834	1,9521	2,9972	1,9089

Supplementary Table S5A (continued)

DS	ID						
15	AS68	11,423324	-5,5318	-3,4157	-1,0130	-0,3490	-0,2003
		**(min)**	**-5,5318**	**-3,4157**	**-3,6028**	**-2,1571**	**-1,5740**
		**(mean neg. dev.)**	**-2,9504**	**-2,0474**	**-1,3797**	**-1,0234**	**-0,8034**
		**(mean pos. dev.)**	**3,3718**	**2,3399**	**1,5767**	**1,1695**	**1,2051**
		**(max)**	**8,0963**	**4,6921**	**3,5749**	**2,9972**	**1,9089**

DS	ID	Experimental deltaBMI	Rel. deviation deltaBMI 1 term model	Rel. deviation deltaBMI 2 terms model	Rel. deviation deltaBMI 3 terms model	Rel. deviation deltaBMI 4 terms model	Rel. deviation deltaBMI 5 terms model
1	AS44	3,493755	-72,56%	-49,94%	-17,23%	-7,51%	2,08%
2	AS45	-4,162065	-194,53%	44,88%	16,33%	-0,46%	-3,10%
3	AS50	5,497006	-61,69%	-34,98%	-20,77%	-30,76%	-27,78%
4	AS51	0,730460	215,20%	335,31%	20,65%	-22,15%	-89,57%
5	AS53	-4,514210	-119,46%	-103,94%	-39,00%	-22,87%	-42,16%
6	AS56	7,136672	-40,78%	-11,28%	13,25%	18,12%	25,27%
7	AS58	4,577500	-39,89%	-36,39%	-78,71%	-30,02%	-28,10%
8	AS60	8,923260	-43,02%	-34,50%	-27,25%	-19,25%	-13,88%
9	AS61	5,119567	52,05%	32,16%	7,07%	-42,13%	-30,74%
10	AS62	5,900277	-4,15%	11,76%	-6,60%	1,85%	-2,88%
11	AS63	3,689796	7,41%	30,92%	62,09%	21,74%	38,29%
12	AS64	5,607585	-59,30%	-33,51%	-20,98%	-8,45%	-9,42%
13	AS65	-1,212121	-236,01%	-195,79%	-294,93%	-159,54%	4,27%
14	AS66	4,996348	54,89%	67,72%	39,07%	59,99%	38,21%
15	AS68	11,423324	-48,43%	-29,90%	-8,87%	-3,06%	-1,75%
		**(min)**	**-236,01%**	**-195,79%**	**-294,93%**	**-159,54%**	**-89,57%**
		**(mean neg. dev.)**	**-83,62%**	**-58,91%**	**-57,15%**	**-31,47%**	**-24,94%**
		**(mean pos. dev.)**	**82,39%**	**87,12%**	**26,41%**	**25,42%**	**21,62%**
		**(max)**	**215,20%**	**335,31%**	**62,09%**	**59,99%**	**38,29%**

	1 term model	2 terms model	3 terms model	4 terms model	5 terms model	
df	3	4	5	6	7	
**AICc**	90,1	81,1	77,2	74,9	77,8	
**σ**	3,9824	2,7067	2,1228	1,7002	1,5389	
**adjusted R^2**	0,1985	0,6297	0,7722	0,8539	0,8803	
**Predictor variables**	Symbiobacterium	Megasphaera<br>Symbiobacterium	Marvinbryantia<br>Megasphaera<br>Symbiobacterium	Blautia<br>Marvinbryantia<br>Megasphaera<br>Symbiobacterium	Blautia<br>Marvinbryantia<br>Megasphaera<br>Oscillibacter<br>Symbiobacterium	
**Indices of predictor variables**	70	43<br>70	58<br>43<br>70	12<br>58<br>43<br>70	12<br>58<br>43<br>17<br>70	
**Terms (estimates)**	-0,4129	1,3943	2,1548	2,3471	2,9371	β_0
	40108,2857	-14712,3712	-13801,3176	385,3526	483,5248	β_1
		56023,4447	-13524,0523	-21318,3744	-20286,0947	β_2
			78001,1437	-27353,0537	-30415,3579	β_3
				84307,6324	-2275,6930	β_4
					130818,0411	β_5

Supplementary Table S5B

Predicted weight loss, calculated as delta BMI, including classification and relative as well as absolute deviations of the predicted weight loss to the observed weight loss. Values are calculated for each patient under the optimal linear regression models with one to five linear terms containing predictor variables from the final set of selected species of Table 6. Predictions for patients, numbered from 1 – 15 were classified as "correct(+)" in case significant weight loss occurred and was predicted, as "correct(-)" in case significant weight loss did not occur and was predicted as not occurring, as "overpredicted" in case a model predicted significant weight loss but it did not occur, and as "underpredicted" in case a model predicted no significant weight loss but significant weight loss occurred . The threshold for a significant weight loss was chosen as 4.8 in case of delta BMI.

Absolute deviations are the differences between predicted and observed BMI.

Relative deviations are absolute deviations divided by observed BMI.

DS	ID	Experimental deltaBMI	Predicted deltaBMI 1 term model	Predicted deltaBMI 2 terms model	Predicted deltaBMI 3 terms model	Predicted deltaBMI 4 terms model	Predicted deltaBMI 5 terms model
1	AS44	3,493755	1,209862	0,556251	1,569926	2,605970	2,722080
2	AS45	-4,162065	2,003930	-4,430295	-4,548929	-4,199125	-4,863081
3	AS50	5,497006	2,348452	3,249720	4,581538	5,365381	5,594149
4	AS51	0,730460	1,520963	1,824863	2,265738	0,436464	0,556101
5	AS53	-4,514210	0,997941	1,143811	-3,825890	-3,480628	-3,546740
6	AS56	7,136672	5,820853	7,733478	8,407433	8,891413	8,757313
7	AS58	4,577500	1,642979	2,382581	2,851883	3,823414	4,001707
8	AS60	8,923260	7,838665	10,406615	7,490986	7,355285	7,475410
9	AS61	5,119567	5,126517	5,546941	5,957365	6,532467	6,160304
10	AS62	5,900277	3,947900	4,426650	5,112201	5,754227	5,572788
11	AS63	3,689796	9,372458	3,704056	3,700322	3,428687	4,072275
12	AS64	5,607585	3,636668	5,004917	5,494150	3,705676	3,647632
13	AS65	-1,212121	2,203936	2,332915	2,968813	0,435141	0,421994
14	AS66	4,996348	1,726790	2,923610	3,602172	4,631671	5,245066
15	AS68	11,423324	7,809050	10,400292	11,579448	11,920629	11,390202

				deltaBMI	threshold:	4,8

DS	ID	Experimental deltaBMI	Classification deltaBMI 1 term model	Classification deltaBMI 2 terms model	Classification deltaBMI 3 terms model	Classification deltaBMI 4 terms model	Classification deltaBMI 5 terms model
1	AS44	3,493755	correct(-)	correct(-)	correct(-)	correct(-)	correct(-)
2	AS45	-4,162065	correct(-)	correct(-)	correct(-)	correct(-)	correct(-)
3	AS50	5,497006	underpredicted	underpredicted	underpredicted	correct(+)	correct(+)
4	AS51	0,730460	correct(-)	correct(-)	correct(-)	correct(-)	correct(-)
5	AS53	-4,514210	correct(-)	correct(-)	correct(-)	correct(-)	correct(-)
6	AS56	7,136672	correct(+)	correct(+)	correct(+)	correct(+)	correct(+)
7	AS58	4,577500	correct(-)	correct(-)	correct(-)	correct(-)	correct(-)
8	AS60	8,923260	correct(+)	correct(+)	correct(+)	correct(+)	correct(+)
9	AS61	5,119567	correct(+)	correct(+)	correct(+)	correct(+)	correct(+)
10	AS62	5,900277	underpredicted	underpredicted	correct(+)	correct(+)	correct(+)
11	AS63	3,689796	overpredicted	correct(-)	correct(-)	correct(-)	correct(-)
12	AS64	5,607585	underpredicted	correct(+)	correct(+)	underpredicted	underpredicted
13	AS65	-1,212121	correct(-)	correct(-)	correct(-)	correct(-)	correct(-)
14	AS66	4,996348	underpredicted	underpredicted	underpredicted	underpredicted	correct(+)
15	AS68	11,423324	correct(+)	correct(+)	correct(+)	correct(+)	correct(+)
		correct(+)	4	5	6	6	7
		correct(-)	6	7	7	7	7
		underpredicted	4	3	2	2	1
		overpredicted	1	0	0	0	0
		sum correct	10	12	13	13	14
		sum incorrect	5	3	2	2	1

DS	ID	Experimental deltaBMI	Abs. deviation deltaBMI 1 term model	Abs. deviation deltaBMI 2 terms model	Abs. deviation deltaBMI 3 terms model	Abs. deviation deltaBMI 4 terms model	Abs. deviation deltaBMI 5 terms model
1	AS44	3,493755	-2,2839	-2,9375	-1,9238	-0,8878	-0,7717
2	AS45	-4,162065	6,1660	-0,2682	-0,3869	-0,0371	-0,7010
3	AS50	5,497006	-3,1486	-2,2473	-0,9155	-0,1316	0,0971
4	AS51	0,730460	0,7905	1,0944	1,5353	-0,2940	-0,1744
5	AS53	-4,514210	5,5122	5,6580	0,6883	1,0336	0,9675
6	AS56	7,136672	-1,3158	0,5968	1,2708	1,7547	1,6206
7	AS58	4,577500	-2,9345	-2,1949	-1,7256	-0,7541	-0,5758
8	AS60	8,923260	-1,0846	1,4834	-1,4323	-1,5680	-1,4479
9	AS61	5,119567	0,0070	0,4274	0,8378	1,4129	1,0407
10	AS62	5,900277	-1,9524	-1,4736	-0,7881	-0,1461	-0,3275
11	AS63	3,689796	5,6827	0,0143	0,0105	-0,2611	0,3825
12	AS64	5,607585	-1,9709	-0,6027	-0,1134	-1,9019	-1,9600
13	AS65	-1,212121	3,4161	3,5450	4,1809	1,6473	1,6341

Supplementary Table S5B (continued)

14	AS66	4,996348	-3,2696	-2,0727	-1,3942	-0,3647	0,2487
15	AS68	11,423324	-3,6143	-1,0230	0,1561	0,4973	-0,0331
		**(min)**	**-3,6143**	**-2,9375**	**-1,9238**	**-1,9019**	**-1,9600**
		**(mean neg. dev.)**	**-2,3972**	**-1,6025**	**-1,0850**	**-0,6346**	**-0,7489**
		**(mean pos. dev.)**	**3,5957**	**1,8313**	**1,2400**	**1,2692**	**0,8559**
		**(max)**	**6,1660**	**5,6580**	**4,1809**	**1,7547**	**1,6341**

**DS**	**ID**	**Experimental deltaBMI**	**Rel. deviation deltaBMI 1 term model**	**Rel. deviation deltaBMI 2 terms model**	**Rel. deviation deltaBMI 3 terms model**	**Rel. deviation deltaBMI 4 terms model**	**Rel. deviation deltaBMI 5 terms model**
1	AS44	3,493755	-65,37%	-84,08%	-55,06%	-25,41%	-22,09%
2	AS45	-4,162065	-148,15%	6,44%	9,29%	0,89%	16,84%
3	AS50	5,497006	-57,28%	-40,88%	-16,65%	-2,39%	1,77%
4	AS51	0,730460	108,22%	149,82%	210,18%	-40,25%	-23,87%
5	AS53	-4,514210	-122,11%	-125,34%	-15,25%	-22,90%	-21,43%
6	AS56	7,136672	-18,44%	8,36%	17,81%	24,59%	22,71%
7	AS58	4,577500	-64,11%	-47,95%	-37,70%	-16,47%	-12,58%
8	AS60	8,923260	-12,15%	16,62%	-16,05%	-17,57%	-16,23%
9	AS61	5,119567	0,14%	8,35%	16,36%	27,60%	20,33%
10	AS62	5,900277	-33,09%	-24,98%	-13,36%	-2,48%	-5,55%
11	AS63	3,689796	154,01%	0,39%	0,29%	-7,08%	10,37%
12	AS64	5,607585	-35,15%	-10,75%	-2,02%	-33,92%	-34,95%
13	AS65	-1,212121	-281,82%	-292,47%	-344,93%	-135,90%	-134,81%
14	AS66	4,996348	-65,44%	-41,49%	-27,90%	-7,30%	4,98%
15	AS68	11,423324	-31,64%	-8,96%	1,37%	4,35%	-0,29%
		**(min)**	**-281,82%**	**-292,47%**	**-344,93%**	**-135,90%**	**-134,81%**
		**(mean neg. dev.)**	**-77,90%**	**-75,21%**	**-58,77%**	**-28,33%**	**-30,20%**
		**(mean pos. dev.)**	**87,46%**	**31,66%**	**42,55%**	**14,36%**	**12,83%**
		**(max)**	**154,01%**	**149,82%**	**210,18%**	**27,60%**	**22,71%**

	**1 term model**	**2 terms model**	**3 terms model**	**4 terms model**	**5 terms model**	
df	3	4	5	6	7	
**AICc**	87,3	78,7	71,9	66,8	72,6	
**σ**	3,6278	2,4994	1,7824	1,2986	1,2922	
**adjusted R^2**	0,3348	0,6843	0,8394	0,9148	0,9156	
**Predictor variables**	Alistipes finegoldii	Alistipes finegoldii<br>Bacteroides caccae	Alistipes finegoldii<br>Bacteroides caccae<br>Roseburia intestinalis	Alistipes finegoldii<br>Bacteroides caccae<br>Bacteroides stercoris<br>Roseburia intestinalis	Alistipes finegoldii<br>Alistipes spHGB5<br>Bacteroides caccae<br>Bacteroides stercoris<br>Roseburia intestinalis	
**Indices of predictor variables**	8	8<br>59	8<br>59<br>12	8<br>59<br>40<br>12	8<br>20<br>59<br>40<br>12	
**Terms (estimates)**	0,3472	1,6915	3,2493	4,5817	5,0939	β_0
	182,8347	220,7382	222,4464	200,8240	144,9347	β_1
		-982,1785	-1155,4981	-1193,2381	157,8646	β_2
			-53,0409	-202,3671	-1298,3441	β_3
				-57,3845	-206,3933	β_4
					-60,7754	β_5

Supplementary Table S6A

Predicted weight loss, calculated as RWL, including classification and relative as well as absolute deviations of the predicted weight loss to the observed weight loss. Values are calculated for each patient under the optimal linear regression models with one to five linear terms containing predictor variables from the final set of selected genera of Table 6. Predictions for patients, numbered from 1 – 15 were classified as "correct(+)" in case significant weight loss occurred and was predicted, as "correct(-)" in case significant weight loss did not occur and was predicted as not occurring, as "overpredicted" in case a model predicted significant weight loss but it did not occur, and as "underpredicted" in case a model predicted no significant weight loss but significant weight loss occurred . The threshold for a significant weight loss was chosen as 10 in case of RWL.

Absolute deviations are the differences between predicted and RWL.

Relative deviations are absolute deviations divided by RWL.

DS	ID	Experimental RWL	Predicted RWL 1 term model	Predicted RWL 2 terms model	Predicted RWL 3 terms model	Predicted RWL 4 terms model	Predicted RWL 5 terms model
1	AS44	7,419621	1,308516	3,184003	5,591291	6,195228	6,838722
2	AS45	-10,017575	9,710818	-13,940128	-11,437047	-10,193966	-9,982516
3	AS50	14,672897	4,547772	8,032874	9,677719	8,700844	9,015824
4	AS51	1,790831	5,102750	7,185145	2,343230	1,787038	0,841849
5	AS53	-10,819672	1,081995	-0,581084	-6,756898	-8,052501	-6,380540
6	AS56	17,835821	10,534620	15,532966	19,220151	19,838543	20,818698
7	AS58	8,203593	6,371269	6,751164	2,670664	6,635655	6,804258
8	AS60	20,636285	12,958976	14,763515	16,125747	17,396813	18,315298
9	AS61	15,848671	20,582147	18,165808	15,460239	10,978208	12,097402
10	AS62	17,401961	14,571843	16,799465	14,516965	15,404342	14,868511
11	AS63	7,463672	9,793000	11,851418	14,274403	11,625550	12,797452
12	AS64	12,247475	5,046119	8,478778	9,958832	11,208526	11,104548
13	AS65	-2,532617	3,256600	2,099283	4,630703	1,710986	-2,100022
14	AS66	14,491525	20,454830	21,975683	18,960576	20,819913	18,731161
15	AS68	25,916667	15,237940	20,260600	25,321887	26,503242	26,788622

RWL threshold: 10

DS	ID	Experimental RWL	Classification RWL 1 term model	Classification RWL 2 terms model	Classification RWL 3 terms model	Classification RWL 4 terms model	Classification RWL 5 terms model
1	AS44	7,419621	correct(-)	correct(-)	correct(-)	correct(-)	correct(-)
2	AS45	-10,017575	correct(-)	correct(-)	correct(-)	correct(-)	correct(-)
3	AS50	14,672897	underpredicted	underpredicted	underpredicted	underpredicted	underpredicted
4	AS51	1,790831	correct(-)	correct(-)	correct(-)	correct(-)	correct(-)
5	AS53	-10,819672	correct(-)	correct(-)	correct(-)	correct(-)	correct(-)
6	AS56	17,835821	correct(+)	correct(+)	correct(+)	correct(+)	correct(+)
7	AS58	8,203593	correct(-)	correct(-)	correct(-)	correct(-)	correct(-)
8	AS60	20,636285	correct(+)	correct(+)	correct(+)	correct(+)	correct(+)
9	AS61	15,848671	correct(+)	correct(+)	correct(+)	correct(+)	correct(+)
10	AS62	17,401961	correct(+)	correct(+)	correct(+)	correct(+)	correct(+)
11	AS63	7,463672	correct(-)	overpredicted	overpredicted	overpredicted	overpredicted
12	AS64	12,247475	underpredicted	underpredicted	underpredicted	correct(+)	correct(+)
13	AS65	-2,532617	correct(-)	correct(-)	correct(-)	correct(-)	correct(-)
14	AS66	14,491525	correct(+)	correct(+)	correct(+)	correct(+)	correct(+)
15	AS68	25,916667	correct(+)	correct(+)	correct(+)	correct(+)	correct(+)
		correct(+)	6	6	6	7	7
		correct(-)	7	6	6	6	6
		underpredicted	2	2	2	1	1
		overpredicted	0	1	1	1	1
		sum correct	13	12	12	13	13
		sum incorrect	2	3	3	2	2

DS	ID	Experimental RWL	Abs. deviation RWL 1 term model	Abs. deviation RWL 2 terms model	Abs. deviation RWL 3 terms model	Abs. deviation RWL 4 terms model	Abs. deviation RWL 5 terms model
1	AS44	7,419621	-6,1111	-4,2356	-1,8283	-1,2244	-0,5809
2	AS45	-10,017575	19,7284	-3,9226	-1,4195	-0,1764	0,0351
3	AS50	14,672897	-10,1251	-6,6400	-4,9952	-5,9721	-5,6571
4	AS51	1,790831	3,3119	5,3943	0,5524	-0,0038	-0,9490
5	AS53	-10,819672	11,9017	10,2386	4,0628	2,7672	4,4391
6	AS56	17,835821	-7,3012	-2,3029	1,3843	2,0027	2,9829
7	AS58	8,203593	-1,8323	-1,4524	-5,5329	-1,5679	-1,3993
8	AS60	20,636285	-7,6773	-5,8728	-4,5105	-3,2395	-2,3210
9	AS61	15,848671	4,7335	2,3171	-0,3884	-4,8705	-3,7513
10	AS62	17,401961	-2,8301	-0,6025	-2,8850	-1,9976	-2,5335
11	AS63	7,463672	2,3293	4,3877	6,8107	4,1619	5,3338
12	AS64	12,247475	-7,2014	-3,7687	-2,2886	-1,0389	-1,1429
13	AS65	-2,532617	5,7892	4,6319	7,1633	4,2436	0,4326
14	AS66	14,491525	5,9633	7,4842	4,4691	6,3284	4,2396

Supplementary Table S6A (continued)

15	AS68	25,916667	-10,6787	-5,6561	-0,5948	0,5866	0,8720
		**(min)**	**-10,6787**	**-6,6400**	**-5,5329**	**-5,9721**	**-5,6571**
		**(mean neg. dev.)**	**-6,7197**	**-3,8282**	**-2,7159**	**-2,2323**	**-2,2919**
		**(mean pos. dev.)**	**7,6796**	**5,7423**	**4,0738**	**3,3484**	**2,6193**
		**(max)**	**19,7284**	**10,2386**	**7,1633**	**6,3284**	**5,3338**

DS	ID	Experimental RWL	Rel. deviation RWL 1 term model	Rel. deviation RWL 2 terms model	Rel. deviation RWL 3 terms model	Rel. deviation RWL 4 terms model	Rel. deviation RWL 5 terms model
1	AS44	7,419621	-82,36%	-57,09%	-24,64%	-16,50%	-7,83%
2	AS45	-10,017575	-196,94%	39,16%	14,17%	1,76%	-0,35%
3	AS50	14,672897	-69,01%	-45,25%	-34,04%	-40,70%	-38,55%
4	AS51	1,790831	184,94%	301,22%	30,85%	-0,21%	-52,99%
5	AS53	-10,819672	-110,00%	-94,63%	-37,55%	-25,58%	-41,03%
6	AS56	17,835821	-40,94%	-12,91%	7,76%	11,23%	16,72%
7	AS58	8,203593	-22,34%	-17,70%	-67,45%	-19,11%	-17,06%
8	AS60	20,636285	-37,20%	-28,46%	-21,86%	-15,70%	-11,25%
9	AS61	15,848671	29,87%	14,62%	-2,45%	-30,73%	-23,67%
10	AS62	17,401961	-16,26%	-3,46%	-16,58%	-11,48%	-14,56%
11	AS63	7,463672	31,21%	58,79%	91,25%	55,76%	71,46%
12	AS64	12,247475	-58,80%	-30,77%	-18,69%	-8,48%	-9,33%
13	AS65	-2,532617	-228,59%	-182,89%	-282,84%	-167,56%	-17,08%
14	AS66	14,491525	41,15%	51,65%	30,84%	43,67%	29,26%
15	AS68	25,916667	-41,20%	-21,82%	-2,29%	2,26%	3,36%
		**(min)**	**-228,59%**	**-182,89%**	**-282,84%**	**-167,56%**	**-52,99%**
		**(mean neg. dev.)**	**-82,15%**	**-49,50%**	**-50,84%**	**-33,61%**	**-21,25%**
		**(mean pos. dev.)**	**71,79%**	**93,09%**	**34,97%**	**22,94%**	**30,20%**
		**(max)**	**184,94%**	**301,22%**	**91,25%**	**55,76%**	**71,46%**

	1 term model	2 terms model	3 terms model	4 terms model	5 terms model	
df	3	4	5	6	7	
**AICc**	114,7	103,9	100,3	101,2	105,9	
**σ**	9,0643	5,7871	4,5898	4,0784	3,9209	
**adjusted R^2**	0,2956	0,7129	0,8194	0,8574	0,8682	
**Predictor variables**	Symbio-bacterium	Megasphaera<br>Symbio-bacterium	Marvinbryantia<br>Megasphaera<br>Symbio-bacterium	Blautia<br>Marvinbryantia<br>Megasphaera<br>Symbio-bacterium	Blautia<br>Marvinbryantia<br>Megasphaera<br>Oscillibacter<br>Symbio-bacterium	
**Indices of predictor variables**	70	43<br>70	58<br>43<br>70	12<br>58<br>43<br>70	12<br>58<br>43<br>17<br>70	
**Terms (estimates)**	-2,5650	1,7245	3,3265	3,6686	4,8010	β_0
	113260,7034	-34920,8953	-29073,0909	685,5863	874,0117	β_1
		151036,5041	-32417,6487	-42446,7954	-40465,5047	β_2
			197333,5067	-57021,0251	-62898,6129	β_3
				208553,4721	-4367,8173	β_4
					297822,5306	β_5

Supplementary Table S6B

Predicted weight loss, calculated as RWL, including classification and relative as well as absolute deviations of the predicted weight loss to the observed weight loss. Values are calculated for each patient under the optimal linear regression models with one to five linear terms containing predictor variables from the final set of selected species of Table 6. Predictions for patients, numbered from 1 – 15 were classified as "correct(+)" in case significant weight loss occurred and was predicted, as "correct(-)" in case significant weight loss did not occur and was predicted as not occurring, as "overpredicted" in case a model predicted significant weight loss but it did not occur, and as "underpredicted" in case a model predicted no significant weight loss but significant weight loss occurred . The threshold for a significant weight loss was chosen as 10 in case of RWL.

Absolute deviations are the differences between predicted and RWL.

Relative deviations are absolute deviations divided by RWL.

DS	ID	Experimental RWL	Predicted RWL 1 term model	Predicted RWL 2 terms model	Predicted RWL 3 terms model	Predicted RWL 4 terms model	Predicted RWL 5 terms model
1	AS44	7,419621	3,358021	1,735744	4,311619	7,119686	5,287959
2	AS45	-10,017575	5,191555	-10,778968	-11,080497	-10,132400	-9,928366
3	AS50	14,672897	5,987070	8,224208	11,608545	13,733050	12,801215
4	AS51	1,790831	4,076365	4,830757	5,951041	0,993021	0,897392
5	AS53	-10,819672	2,868688	3,230826	-9,398169	-8,462374	-8,706831
6	AS56	17,835821	14,004983	18,752444	20,465023	21,776791	20,751212
7	AS58	8,203593	4,358105	6,193967	7,386489	10,019700	9,686376
8	AS60	20,636285	18,664190	25,038256	17,629033	17,261237	17,162297
9	AS61	15,848671	12,401734	13,445355	14,488254	16,046995	17,370635
10	AS62	17,401961	9,680260	10,868653	12,610704	14,350831	16,041907
11	AS63	7,463672	22,205780	8,136136	8,126581	7,390345	7,203286
12	AS64	12,247475	8,961612	12,357860	13,601029	8,753592	8,761065
13	AS65	-2,532617	5,653377	5,973593	7,589467	0,722263	0,860035
14	AS66	14,491525	4,551628	7,522363	9,246657	12,036983	13,874751
15	AS68	25,916667	18,595809	25,027688	28,024077	28,948802	28,496656

DS	ID	Experimental RWL	Classification RWL 1 term model	Classification RWL 2 terms model	Classification RWL 3 terms model	Classification RWL 4 terms model	Classification RWL 5 terms model
					RWL	threshold:	10
1	AS44	7,419621	correct(-)	correct(-)	correct(-)	correct(-)	correct(-)
2	AS45	-10,017575	correct(-)	correct(-)	correct(-)	correct(-)	correct(-)
3	AS50	14,672897	underpredicted	underpredicted	correct(+)	correct(+)	correct(+)
4	AS51	1,790831	correct(-)	correct(-)	correct(-)	correct(-)	correct(-)
5	AS53	-10,819672	correct(-)	correct(-)	correct(-)	correct(-)	correct(-)
6	AS56	17,835821	correct(+)	correct(+)	correct(+)	correct(+)	correct(+)
7	AS58	8,203593	correct(-)	correct(-)	correct(-)	overpredicted	correct(-)
8	AS60	20,636285	correct(+)	correct(+)	correct(+)	correct(+)	correct(+)
9	AS61	15,848671	correct(+)	correct(+)	correct(+)	correct(+)	correct(+)
10	AS62	17,401961	underpredicted	correct(+)	correct(+)	correct(+)	correct(+)
11	AS63	7,463672	overpredicted	correct(-)	correct(-)	correct(-)	correct(-)
12	AS64	12,247475	underpredicted	correct(+)	correct(+)	underpredicted	underpredicted
13	AS65	-2,532617	correct(-)	correct(-)	correct(-)	correct(-)	correct(-)
14	AS66	14,491525	underpredicted	underpredicted	underpredicted	correct(+)	correct(+)
15	AS68	25,916667	correct(+)	correct(+)	correct(+)	correct(+)	correct(+)
		correct(+)	4	6	7	7	7
		correct(-)	6	7	7	6	7
		underpredicted	4	2	1	1	1
		overpredicted	1	0	0	1	0
		sum correct	10	13	14	13	14
		sum incorrect	5	2	1	2	1

DS	ID	Experimental RWL	Abs. deviation RWL 1 term model	Abs. deviation RWL 2 terms model	Abs. deviation RWL 3 terms model	Abs. deviation RWL 4 terms model	Abs. deviation RWL 5 terms model
1	AS44	7,419621	-4,0616	-5,6839	-3,1080	-0,2999	-2,1317
2	AS45	-10,017575	15,2091	-0,7614	-1,0629	-0,1148	0,0892
3	AS50	14,672897	-8,6858	-6,4487	-3,0644	-0,9398	-1,8717
4	AS51	1,790831	2,2855	3,0399	4,1602	-0,7978	-0,8934
5	AS53	-10,819672	13,6884	14,0505	1,4215	2,3573	2,1128
6	AS56	17,835821	-3,8308	0,9166	2,6292	3,9410	2,9154
7	AS58	8,203593	-3,8455	-2,0096	-0,8171	1,8161	1,4828
8	AS60	20,636285	-1,9721	4,4020	-3,0073	-3,3750	-3,4740
9	AS61	15,848671	-3,4469	-2,4033	-1,3604	0,1983	1,5220
10	AS62	17,401961	-7,7217	-6,5333	-4,7913	-3,0511	-1,3601
11	AS63	7,463672	14,7421	0,6725	0,6629	-0,0733	-0,2604
12	AS64	12,247475	-3,2859	0,1104	1,3536	-3,4939	-3,4864
13	AS65	-2,532617	8,1860	8,5062	10,1221	3,2549	3,3927

Supplementary Table S6B (continued)

14	AS66	14,491525	-9,9399	-6,9692	-5,2449	-2,4545	-0,6168
15	AS68	25,916667	-7,3209	-0,8890	2,1074	3,0321	2,5800
		**(min)**	**-9,9399**	**-6,9692**	**-5,2449**	**-3,4939**	**-3,4864**
		**(mean neg. dev.)**	**-5,4111**	**-3,9623**	**-2,8070**	**-1,6223**	**-1,7618**
		**(mean pos. dev.)**	**10,8222**	**4,5283**	**3,2081**	**2,4333**	**2,0135**
		**(max)**	**15,2091**	**14,0505**	**10,1221**	**3,9410**	**3,3927**

DS	ID	Experimental RWL	Rel. deviation RWL 1 term model	Rel. deviation RWL 2 terms model	Rel. deviation RWL 3 terms model	Rel. deviation RWL 4 terms model	Rel. deviation RWL 5 terms model
1	AS44	7,419621	-54,74%	-76,61%	-41,89%	-4,04%	-28,73%
2	AS45	-10,017575	-151,82%	7,60%	10,61%	1,15%	-0,89%
3	AS50	14,672897	-59,20%	-43,95%	-20,88%	-6,41%	-12,76%
4	AS51	1,790831	127,62%	169,75%	232,31%	-44,55%	-49,89%
5	AS53	-10,819672	-126,51%	-129,86%	-13,14%	-21,79%	-19,53%
6	AS56	17,835821	-21,48%	5,14%	14,74%	22,10%	16,35%
7	AS58	8,203593	-46,88%	-24,50%	-9,96%	22,14%	18,07%
8	AS60	20,636285	-9,56%	21,33%	-14,57%	-16,35%	-16,83%
9	AS61	15,848671	-21,75%	-15,16%	-8,58%	1,25%	9,60%
10	AS62	17,401961	-44,37%	-37,54%	-27,53%	-17,53%	-7,82%
11	AS63	7,463672	197,52%	9,01%	8,88%	-0,98%	-3,49%
12	AS64	12,247475	-26,83%	0,90%	11,05%	-28,53%	-28,47%
13	AS65	-2,532617	-323,22%	-335,87%	-399,67%	-128,52%	-133,96%
14	AS66	14,491525	-68,59%	-48,09%	-36,19%	-16,94%	-4,26%
15	AS68	25,916667	-28,25%	-3,43%	8,13%	11,70%	9,95%
		**(min)**	**-323,22%**	**-335,87%**	**-399,67%**	**-128,52%**	**-133,96%**
		**(mean neg. dev.)**	**-75,63%**	**-79,45%**	**-63,60%**	**-28,56%**	**-27,87%**
		**(mean pos. dev.)**	**162,57%**	**35,62%**	**47,62%**	**11,67%**	**13,49%**
		**(max)**	**197,52%**	**169,75%**	**232,31%**	**22,14%**	**18,07%**

	1 term model	2 terms model	3 terms model	4 terms model	5 terms model	
df	3	4	5	6	7	
AICc	114,7	106,4	99,4	91	95,9	
σ	9,0649	6,2983	4,4478	2,9069	2,8091	
adjusted R^2	0,2955	0,6599	0,8304	0,9273	0,9323	
Predictor variables	Alistipes finegoldii	Alistipes finegoldii<br>Bacteroides caccae	Alistipes finegoldii<br>Bacteroides caccae<br>Roseburia intestinalis	Alistipes finegoldii<br>Bacteroides caccae<br>Bacteroides stercoris<br>Roseburia intestinalis	Alistipes finegoldii<br>Bacteroides caccae<br>Bacteroides stercoris<br>Ethanolig. harbinense<br>Roseburia intestinalis	
Indices of predictor variables	8	8<br>59	8<br>59<br>12	8<br>59<br>40<br>12	8<br>59<br>40<br>89<br>12	
Terms (estimates)	1,3661	4,7029	8,6615	12,2728	9,8820	β_0
	422,1726	516,2542	520,5949	461,9901	445,1752	β_1
		-2437,8965	-2878,3336	-2980,6231	-2863,9291	β_2
			-134,7868	-548,4907	-462,5387	β_3
				-146,5595	3366,6821	β_4
					-135,9567	β_5
